# Combined transcriptomic and metabolomic analysis revealed that pH changes affected the expression of carbohydrate and ribosome biogenesis-related genes in *Aspergillus niger* SICU-33

**DOI:** 10.3389/fmicb.2024.1389268

**Published:** 2024-06-19

**Authors:** Runji Zhang, Yulan Chen, Wenxian Wang, Juan Chen, Dongyang Liu, Lingzi Zhang, Quanju Xiang, Ke Zhao, Menggen Ma, Xiumei Yu, Qiang Chen, Petri Penttinen, Yunfu Gu

**Affiliations:** ^1^Department of Microbiology, College of Resources, Sichuan Agricultural University, Chengdu, China; ^2^Liangshan Tobacco Corporation of Sichuan Province, Xichang, China

**Keywords:** pH, *Aspergillus niger*, transcriptomics, metabolomics, carbohydrate metabolism, ribosome biogenesis

## Abstract

The process of carbohydrate metabolism and genetic information transfer is an important part of the study on the effects of the external environment on microbial growth and development. As one of the most significant environmental parameters, pH has an important effect on mycelial growth. In this study, the effects of environmental pH on the growth and nutrient composition of *Aspergillus niger* (*A. niger*) filaments were determined. The pH values of the medium were 5, 7, and 9, respectively, and the molecular mechanism was further investigated by transcriptomics and metabolomics methods. The results showed that pH 5 and 9 significantly inhibited filament growth and polysaccharide accumulation of *A. niger*. Further, the mycelium biomass of *A. niger* and the crude polysaccharide content was higher when the medium's pH was 7. The DEGs related to ribosome biogenesis were the most abundant, and the downregulated expression of genes encoding XRN1, RRM, and RIO1 affected protein translation, modification, and carbohydrate metabolism in fungi. The dynamic changes of pargyline and choline were in response to the oxidative metabolism of *A. niger* SICU-33. The ribophorin_I enzymes and DL-lactate may be important substances related to pH changes during carbohydrate metabolism of *A.niger* SICU-33. The results of this study provide useful transcriptomic and metabolomic information for further analyzing the bioinformatic characteristics of *A. niger* and improving the application in ecological agricultural fermentation.

## 1 Introduction

Barley (*Hordeum*) is the fourth leading cereal crop around the world (Dawson et al., 2015), and highland barley (*Hordeum vulgare* L. var. *nudum* hook. f, HB), belonging to the genus *Hordeum* in the family Gramineae, is a grain crop that grows on the plateau under poor soil conditions, distributed mainly in the Qinghai-Tibet Plateau, China (Obadi et al., 2021; Xue et al., 2023). It is the main food for local residents and raw material for related food production and processing (Zeng et al., 2018). At present, the research related to highland barley mainly focuses on food processing, genetic analysis of germplasm resources, determination and analysis of nutrient composition, and the relationship between highland barley cultivation and soil properties (Hadgu et al., 2009; Dou et al., 2021; Nie et al., 2022). Therefore, the isolation of related microorganisms from the soil environment of highland barley will enrich the study of the microbial ecology of highland barley habitat.

Fungi are widely distributed and grow in almost all habitats, including extreme deserts or areas with high salt concentrations, deep sea areas, and other environments (Hawksworth, 2001; Basu et al., 2015; Coleine et al., 2022). In the environment, fungi obtain nutrients by secreting enzymes and absorbing the released molecules (Hawksworth, 2001; Basu et al., 2015). *Aspergillus niger* is a type of fungus that possesses strong metabolic capacity for hydrolyzing carbohydrates and producing proteins and organic acids, secreting a wide variety of different enzymes that allow them to release different nutrients such as biopolymers used in industrial fermentation (Pel et al., 2007; Hossain et al., 2019; Marzo et al., 2019). The extracellular enzymes produced by *A. niger* can degrade various organic materials, decompose soil components, and regulate carbon and nutrient balances, such as β-glucosidase hydrolyzes cellulose disaccharide (Lammirato et al., 2011; Kadri et al., 2017). Because of its enzyme production capacity and high safety, *A. niger* has been widely used in industrial biotechnology (Schuster et al., 2002; Abdel-Azeem et al., 2019). It has been reported that *A. niger* can be used in industry to produce hetero-peptides, such as cyclic deposited peptides and itaconic acid (IA), which are widely used in coatings and synthetic resins (Li and van Luijk, 2011; Boecker et al., 2018). Similarly, *A. niger* has also been used in the commercial production of organic acids, e.g., citric and gluconic acids, and industrial proteins, e.g., fungal enzymes and heterologous proteins (Andersen et al., 2008; Krijgsheld et al., 2013; Xie et al., 2018).

In agricultural production, fungi perform various tasks, such as fixing nitrogen, synthesizing hormones, controlling roots, and withstanding drought. They are also crucial for the stability of soil organic matter and the breakdown of residue, and both biotic and abiotic variables can influence their activity (Qiu et al., 2018; Devi et al., 2020). In practical applications, environmental conditions such as nutrient availability, temperature, relative humidity, water activity, and pH are key factors for fungal growth (Silveira et al., 2019; Tai et al., 2020). Previous research has shown that many fungi can grow across a wide pH range and express specific genes depending on the pH of the environment (Peñalva and Arst, 2002; Manteau et al., 2003). The pH of the environment is a crucial indicator of cell activity. It primarily affects growth, morphogenesis, membrane and cell wall stability, and protein function (Prusky and Sionov, 2021; Koza et al., 2022). However, most studies on the response mechanism of *A. niger* to environmental factors mainly involve industrial, such as the bio adsorption of nano-plastics by *A. niger* (Kadri et al., 2017), citric acid production in industry, and corrosion of metal materials by *A. niger* (Qu et al., 2015; Tong et al., 2019). There are few reports on the effect of pH on the growth and metabolism of *A. niger* in agricultural production. Therefore, it is of great significance to explore the influencing mechanism of environmental pH on the growth and metabolism of *A. niger* to promote its active application in agriculture, such as screening PGPR strains, the production of new biological organic fertilizers, and soil improvement.

Until now, transcriptome and metabolome analysis techniques have been rapidly developed in fungal research (Takano et al., 2019; Huang, 2020; Ma et al., 2021). However, the molecular mechanism of nucleic acid and carbohydrate metabolism of *A. niger* affected by pH is still limited. It is necessary to perform high-throughput investigations of variations in both transcription and expression of genes involved in the changes of nucleic acid and carbohydrate metabolism of *A. niger* in the application of agricultural production. Therefore, the research combined with transcriptomics and metabolomics analysis, and functional gene annotation genes and metabolic pathways were analyzed according to Gene Ontology (GO) and Kyoto Encyclopedia of Genes and Genomes (KEGG) analysis; we discussed the molecular regulatory mechanism of the metabolic process changes of *A. niger* SICU-33 under the influence of different pH. The goals of our study were :(1) to reveal the molecular mechanism of pH change on the expression of genes related to nucleic acid and carbohydrate metabolism in *A. niger*; (2) to characterize the effects of pH changes on the mycelial biomass and polysaccharide content of *A. niger*; and (3) to provide experimental basis for the development of the application of *A. niger* in agroecology and to further study its molecular physiological mechanism under changed relevant environmental conditions.

## 2 Materials and methods

### 2.1 Soil samples collection

Samples collection methods are as follows: the isolation details of rhizosphere soil fungi have been referred to in the article of Sun et al. (2022). In 2020, the rhizosphere soil samples from highland barley of Lazi (LZ), Cuomei (CM), Jiangzi (JZ), Longzi4ling (LZ4), Longzi6ling (LZ6), Ali, Zangqing 2000 (ZQ), and Langkazi (LKZ) were collected from eight sites in the Tibetan Plateau for this investigation, and soil fractions from the rhizosphere were brushed and mixed into one sample. The samples were transported in a refrigerated box to the lab, where they were kept at −20°C until the microbes were isolated and purified (Jin et al., 2021).

### 2.2 Culture and isolation of strains

The rhizosphere soil samples were cultured in a PDA liquid medium, and an appropriate amount of sample suspension was added to the surface of the solid medium. They were cultured at 28°C for 3–5 days using the dilution spread plate method. A single colony was selected to be transferred to the new PDA medium for purification, and the pollution-free and well-growing strains were obtained as the target strains (Mahadevamurthy et al., 2016). The plant growth promotion (PGP) potential characteristics of the strains were preliminarily evaluated by the following methods: by testing the IAA production (Glickmann and Dessaux, 1995), isolates were grown in liquid PDA medium (with 2 mg/ml l-tryptophan) at 28°C for 3–5 days in a shaker (150 rpm) pathogen resistance; then cultures were centrifuged at 10,000 *g* for 30 min and 1 ml the supernatant was mixed with 2 ml Salkowski reagent (1 ml of 0.5 M FeCl_3_ and 49 ml of 35% HClO_4_); finally, the IAA concentration was measured at 530 nm after 30 min reaction at room temperature. The yield of siderophore production was performed using the chrome azurol S (CAS) medium, in which the isolated strain was cultured at 28°C for 3 days, and an orange halo was formed around the colony, indicating that siderophore was clearly produced (Schwyn and Neilands, 1987). The phosphorus-solubilizing activity of the isolated strain was determined by the following method (Chen et al., 2022a), in which the strain was inoculated on Pikovskaya's agar and cultured at 28°C for 5 days. Clear halos appeared around the colony, which was considered to have a phosphorus-solubilizing effect. In addition, cellulose degradation was measured as described earlier (Teather and Wood, 1982). All the above measurements included three replicates.

**Figure 1:**
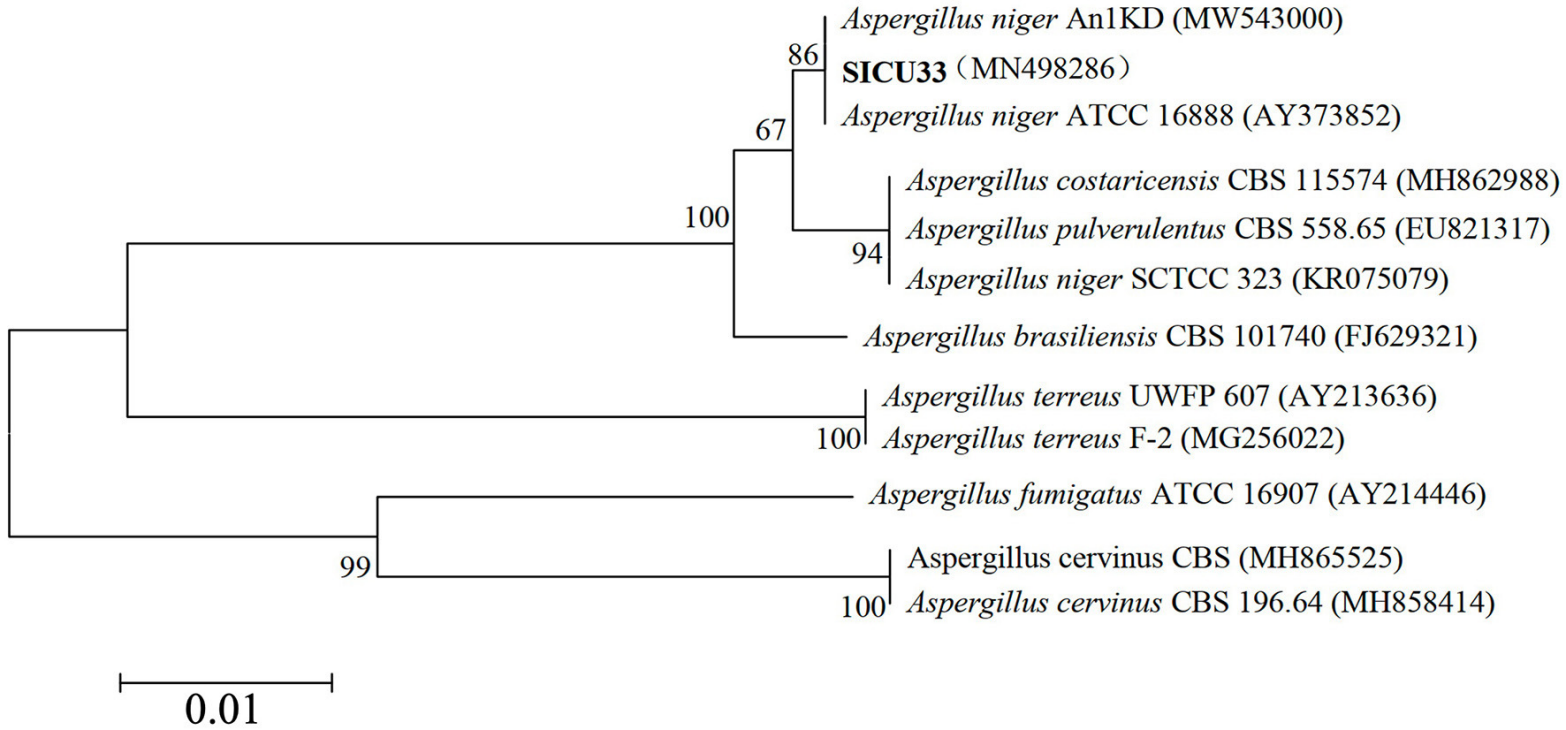
Phylogenetic tree of the ITS gene sequence of SICU-33.

Following the above methods, we obtained highly effective strains that were primed ITS1 (5′-TCCGTAGGTGAACCTGCGG-3′) and ITS4 (5′-TCCTCCGCTTATTGATATGC-3′) amplified and sequenced ITS gene fragments, and the phylogenetic tree of SICU-33 was constructed after BLAST in NCBI (Figure 1). Its sequences were deposited in the GenBank of NCBI (https://www.ncbi.nlm.nih.gov/) under the accession number MN498286. Then, the conventional PDA liquid culture medium was prepared, and the target pH (5, 7, and 9) was reached by adding 1 mol/L of sodium hydroxide and hydrochloric acid solutions. The strain *A. niger* SICU-33 was activated using a PDA medium at 28°C for 3 days, cut off with a 5 mm hole punch, and inoculated in a liquid medium with different pH. After incubating in a constant temperature oscillating incubator at 28°C, 140 r/min for 3 days to the stable stage, the mycelium was filtered with filter paper, drained of water, and weighed. The pH of the medium was continuously measured to ensure that the pH was maintained at a fixed value during this time. After the liquid medium formed the membrane, the samples were collected, dried with filter paper, quickly frozen with liquid nitrogen, and stored at −80°C for extraction and determination of polysaccharides. The polysaccharide content was determined using the sulfuric acid-phenol method (Huang, 2020). All the analyses were done in three replicates.

### 2.3 RNA extraction, cDNA library construction, and sequencing

RNA was extracted using the Trizol Reagent (Invitrogen Life Technologies, USA). The concentration and quality of extracted RNA were determined using a NanoDrop spectrophotometer (NanoDrop Technologies; Thermo Fisher Scientific, Inc., Wilmington, DE, USA). The integrity of the extracted RNA was assessed in 1% agarose by electrophoresis.

Sequencing libraries were generated using the TruSeq RNA Sample Preparation Kit (Illumina, San Diego, CA, USA) (Jain et al., 2020). Briefly, mRNA was purified from 3 μg RNA using poly-T-oligo-attached magnetic beads (Ozsolak and Milos, 2011). Fragmentation was performed using divalent cations under elevated temperature in an Illumina proprietary fragmentation buffer. First-strand cDNA was synthesized using random oligonucleotides and SuperScript II. Second-strand cDNA was synthesized using DNA Polymerase I and RNase H. Remaining overhangs were converted into blunt ends via exonuclease/polymerase activities, and the enzymes were removed. After acetylation of the 3′ ends of the DNA fragments, Illumina PE adapter oligonucleotides were ligated to prepare for hybridization (Wang et al., 2009). To select cDNA fragments of the preferred 200 bp in length, the library fragments were purified using the AMPure XP system (Beckman Coulter, Beverly, CA, USA). DNA fragments with ligated adaptors on both ends were selectively enriched using Illumina PCR Primer Cocktail in a 15-cycle PCR reaction. Products were purified using the AMPure XP system and quantified using the Agilent high-sensitivity DNA assay on a Bioanalyzer 2100 system (Agilent). The sequencing library was sequenced on Illumina Hiseq sequencer at Shanghai Personal Biotechnology Co. Ltd, China. The RNA-seq raw data were submitted to the National Center for Biotechnology Information (NCBI) database (https://www.ncbi.nlm.nih.gov/sra/; accession number PRJNA880770).

### 2.4 Transcriptome analysis

Raw data (raw reads) in Fastq format were first processed through in-house Perl scripts. In this step, clean data (clean reads) were obtained by removing reads containing adapter, poly-N, and low-quality reads. At the same time, Q20, Q30, and GC content of the clean data were calculated. All the downstream analyses were based on the clean data assembled using the Trinity (v2.1.1) software with high quality to construct transcript and unigene sequences. High-quality clean reads were aligned to the assembled transcriptome using HISAT2 (http://ccb.jhu.edu/software/hisat2/index.shtml); the criteria for data filtering included the use of Cutadapt to remove sequences with 3′ end-band connectors and the removal of reads with an average mass score below Q20. The reference genome (*Aspergillus niger*. SICU_33.scaffolds.fna) for this study was derived from whole genome sequencing of SICU_33. In the differential expression analysis using the DESeq R package (version 1.16.1) (Wang et al., 2010; Love et al., 2014), genes were considered differentially expressed when |log_2_(Fold Change)| ≥ 1 and *p* < 0.05. *p*-values were adjusted for multiple testing as described earlier (Mi et al., 2019).

Transcripts were functionally annotated against Universal Protein Knowledgebase 261 (UniProt) (Apweiler et al., 2004), Gene Ontology (GO) (Ashburner et al., 2000), eggNOG (Huerta-Cepas et al., 2016), and Kyoto Encyclopedia of Genes and Genomes (KEGG) databases (Kanehisa et al., 2004) using BLAST (Altschul et al., 1997). GO enrichment analysis by Blast2GO software (Minor Release 5.2.5, default parameter) was performed on the annotated genes. The KEGG pathway enrichment analysis of differentially expressed genes by KAAS (v 2.1, BBH) was performed with the KEGG biological pathways database (http://www.genome.jp) to understand the function of the differentially expressed genes. KEGG orthology (KO) analysis of unigenes was performed using the KOBAS 2.0 web server (http://kobas.cbi.pku.edu.cn/) (Chen et al., 2011).

### 2.5 LC-MS analysis of metabolites

The samples were stored at −80°C for metabolomics analysis. After the samples were thawed slowly at 4°C, 80 mg of samples were taken and added to pre-cooled methanol/acetonitrile/aqueous solution (2:2:1, V/V), quickly frozen in liquid nitrogen and ground into fine powder. After adding 1,000 μl methanol/acetonitrile/H_2_O (2:2:1, V/V/V), the mixture was centrifuged for 15 min at 14,000 *g* and 4°C. The supernatant was collected and dried in a vacuum centrifuge. For LC-MS analysis, the samples were dissolved in 100 μl acetonitrile/water (1:1, V/V).

LC-MS analysis was performed using a 1,290 Infinity LC UHPLC (Agilent Technologies) coupled to an AB Sciex TripleTOF 6600 quadrupole time-of-flight mass spectrometer in Shanghai Applied Protein Technology Co., Ltd, Shanghai, China. The metabolites were determined using both positive and negative mode electrospray injection (ESI). The LC flow rate was 0.3 ml/min at 25°C. Hydrophilic interaction chromatography (HILIC) separation was performed using a 2.1 mm × 100 mm ACQUIY UPLC BEH 1.7 μm column (Waters, Ireland). The mobile phase included 25 mM ammonium acetate and 25 mM ammonium hydroxide in water (solvent A) and acetonitrile (solvent B). Prior to injecting a sample, the column was equilibrated at 95% B for 5 min. The metabolites in 2 μl aliquot of a sample were eluted with 95% B for 0–0.5 min, a linear gradient to 65% B in 0.5–7 min, followed by a gradient to 40% B in 7–8 min, keeping 40% B in 8–9 min, followed by a gradient to 95% B in 9–9.1 min, and 95% B maintained for 9.1–12 min. During the analysis process, the samples were placed in a 4°C automatic injector and analyzed continuously in random order.

The ESI source conditions were set as follows: ion source gas1 as 60, gas2 as 60, curtain gas 30, source temperature 600°C, and ion spray voltage floating ±5,500 V. In the MS-only acquisition, spectra were acquired over the *m*/*z* range of 60–1,000 Da, and the accumulation time for a TOF MS scan was 0.20 s/spectra. In the auto MS/MS acquisition, spectra were acquired over the *m*/*z* range of 25–1,000 Da, and the accumulation time for a product ion scan was 0.05 s/spectra. The product ion spectra were acquired using information-dependent acquisition (IDA) with high sensitivity mode. The parameters were set as follows: the collision energy was fixed at 35 V with ±15 eV, declustering potential was 60 V in the positive mode and −60 V in the negative mode, isotopes within 4 Da were excluded, and 10 candidate ions per cycle were monitored. After preprocessing the original data, peak alignment, peak extraction, normalization, deconvolution, and compound identification were done using XCMS software (Smith and Want, 2006).

### 2.6 Metabolite data analysis

Differentially expressed metabolites were determined using a *t*-test, fold change (FC) analysis, and partial least squares discriminant analysis (PLS-DA) (Chen et al., 2022b). Differences in metabolomes were visualized using principal component analysis (PCA). In this study, the ion identification data in positive mode were selected. Metabolites were considered differentially expressed metabolites (DEMs) when variable importance of projection (VIP) in the PLS-DA model ≥1, fold-change ≥1.5 or ≤ 0.67, and *p*-value < 0.05. In this study, the KEGG database was used to annotate differential metabolites and classify their metabolic pathways.

### 2.7 qRT-PCR validation of RNA-Seq data

To validate the reliability of the RNA-seq data, the expression levels of 19 differentially expressed genes were assessed using quantitative real-time PCR (qRT-PCR) on a CFX96 Real-Time System (Bio-Rad, California, USA) with SYBR green as the fluorescent dye according to the manufacturer's protocol (Sharma, 2016). Real-Time PCR System with a final volume of 10 μl containing 3 μl of cDNA template, 5 μl of SsoFast™ EvaGreen^®^ Supermix (Bio-Rad, USA), 0.2 μl of each forward and reverse primer (Supplementary Table S1), and 1.6 μl of RNase-free water. The qRT-PCR reaction procedure: 94°C 5 min; 94°C 1 min, 53°C 1 min, 72°C 1 min, 40 cycles; 72°C 10 min. The validation included three biological replicates with three technical replicates and ITS as the internal control gene. Genes were considered differentially expressed at *p* < 0.05.

### 2.8 Statistical analysis

Statistical analysis was performed using IBM SPSS Statistics version 26 (IBM Corporation, New York, USA). Data were presented as mean ± standard deviation (SD). The significant differences among groups were analyzed using one-way ANOVA and the Duncan test to determine the significant differences based on *p* < 0.05.

## 3 Results

### 3.1 Effect of pH on strain growth

Different pH conditions (pH 5, pH 7, and pH 9) affected the growth of *A. niger* SICU-33; the biomass of the strains increased first and then decreased in the liquid medium. It can be seen from Table 1 that the strain grew well at pH 7. Both pH 5 and pH 9 led to the reduction of mycelial biomass and inhibited the growth of *A. niger* SICU-33; pH 7 was the most favorable culture condition for this strain. In addition, the polysaccharide content of *A. niger* strain SICU-33 was the highest at pH 7 (Table 1).

**Table 1:** The mycelium biomass, growth rate, and polysaccharide content of the *Aspergillus niger* under different pH conditions (pH 5, pH 7, and pH 9).

**Sample**	**Mycelium biomass (g)**	**Polysaccharide (%)**
pH 5	2.93 ± 0.03b	2.58 ± 0.32c
pH 7	3.67 ± 0.02a	3.49 ± 0.20a
pH 9	3.28 ± 0.01a	3.05 ± 0.15b

Data are presented as average ± SD (n = 3). Different superscript letters a, b, and c in the same row denote statistically significant differences (p < 0.05).

### 3.2 Gene-expression description under different pH

**Figure 2:**
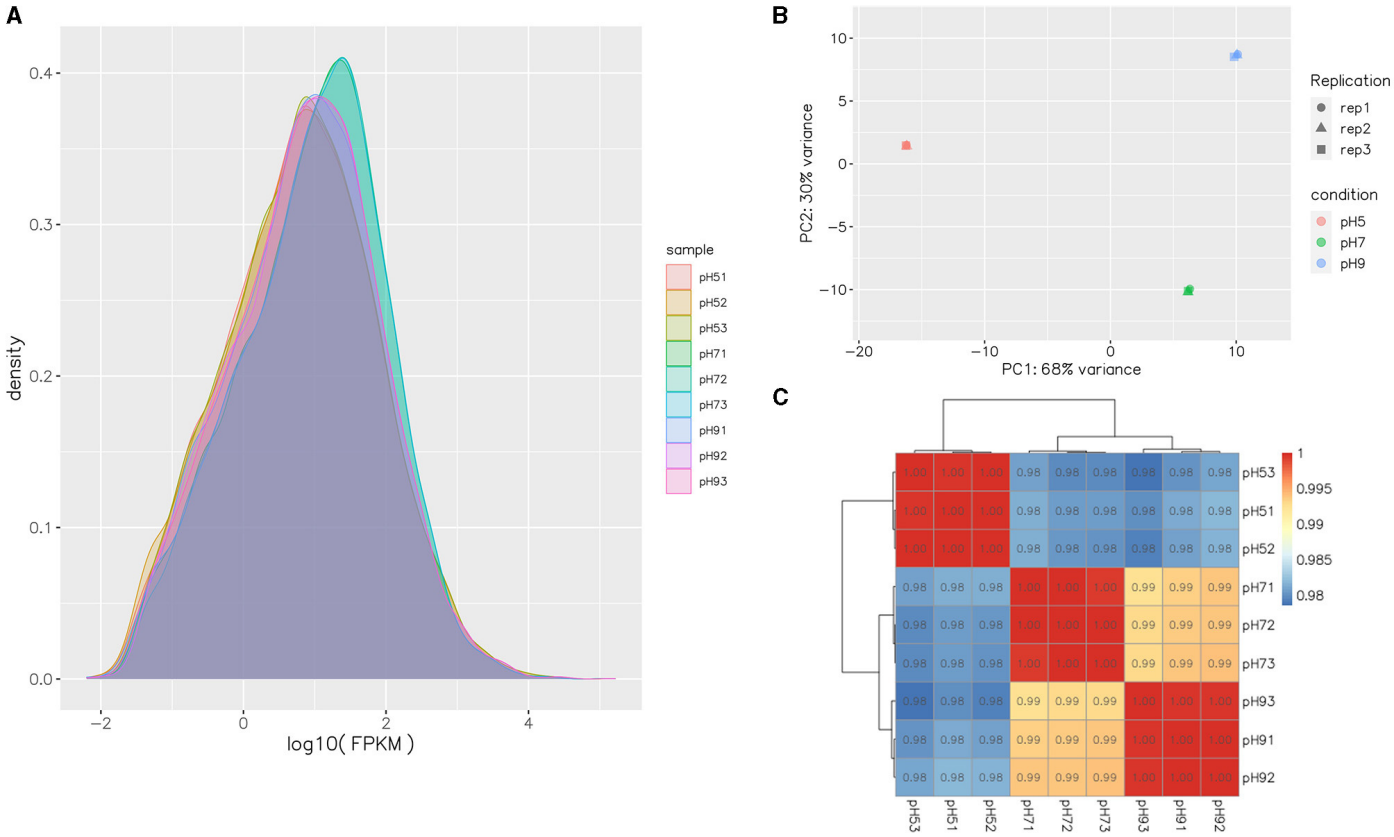
Gene expression FPKM density map of transcripts of *Aspergillus niger* SICU-33 at different pH **(A)**; Principal Components Analysis (PCA) of samples at different pH **(B)**; Pearson correlation test of gene expression levels between samples **(C)**.

After Q20 screening, ~94% of the reads in the cDNA libraries per sample passed the quality filtering, and close to 98% of the quality filtered reads were mapped to *Aspergillus niger*. SICU_33.scaffolds.fna reference genome (Table 2). Approximately 97.57%−97.87% of clean reads were mapped to the reference genome of *A. niger*, 3.14%−7.91% of reads were mapped to multiple positions, and 78.20%−81.11% were mapped to gene, more than 98% of those to exons (Table 2). After different pH treatments, the gene expression density map of *A. niger* SICU-33 showed similar trends in gene abundance and gene expression density (Figure 2A). In addition, PCA analysis (Figure 2B) and correlation heat map (Figure 2C) also showed that there were similarities between repeats of the same treatment and obvious differences between treatments, suggesting that the data could be analyzed for differential expression.

**Table 2:** Statistics of transcriptome sequencing of *Aspergillus niger* SICU-33 under different pH, i.e., pH 5, pH 7, and pH 9, respectively.

**Sample**	**Raw reads**	**Clean reads**	**Clean reads (%)**	**Total mapped**	**Multiple mapped**	**Mapped to gene**	**Mapped to exons**
pH51	48,681,446	45,469,224	93.40%	44,468,066 (97.80%)	3.14%	81.11%	98.75%
pH52	55,126,132	51,699,122	93.78%	50,444,361 (97.57%)	3.64%	81.09%	98.77%
pH53	47,869,964	44,809,794	93.60%	43,765,568 (97.67%)	3.17%	80.87%	98.75%
pH71	46,105,010	43,238,062	93.78%	42,309,522 (97.85%)	3.42%	80.65%	98.91%
pH72	44,174,234	41,511,620	93.97%	40,624,565 (97.86%)	3.71%	80.37%	98.91%
pH73	53,079,524	49,760,440	93.74%	48,639,252 (97.75%)	3.87%	80.28%	98.90%
pH91	56,249,262	52,603,422	93.51%	51,435,628 (97.78%)	7.91%	78.20%	98.88%
pH92	43,841,744	41,169,696	93.90%	40,293,614 (97.87%)	6.78%	78.95%	98.88%
pH93	51,008,940	47,931,428	93.96%	46,900,660 (97.85%)	7.75%	78.96%	98.88%

**Figure 3:**
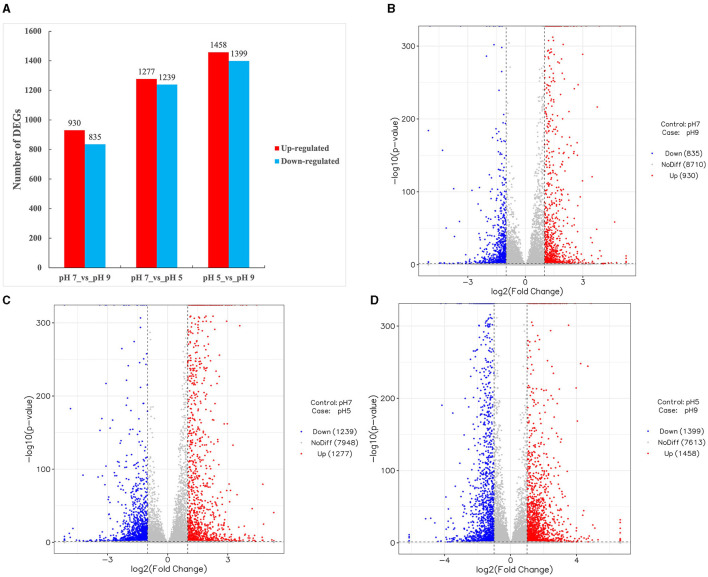
Differentially expressed genes (DEGs) identified among three groups of *Aspergillus niger* SICU-33 samples, i.e., pH 5, pH 7, and pH 9, respectively **(A)**, showing the volcano plots of DEGs between pH 7 vs. pH 9 **(B)**, pH 7 vs. pH 5 **(C)**, and pH 5 vs. pH 9 **(D)**, respectively. DEGs are determined based on |log_2_(Fold Change)| ≥1 and *p* < 0.05.

The expression of genes identified in all three groups of *A. niger* SICU-33 was normalized based on the group of pH 7, with the DEGs identified based on |log_2_(Fold Change) | ≥1 and *p* < 0.05 (Figure 3A). Among the differentially expressed genes (DEGs), 1,277 were upregulated, 1,239 were downregulated in the pH 5 medium compared with pH 7 (Figure 3C), 930 were upregulated, and 835 downregulated at pH 9 compared with pH 7 (Figure 3B), and a total of 2,897 DEGs (1,458 upregulated and 1,399 downregulated) were identified between the pH 5 and pH 9 (Figure 3D). A large number of identified DEGs indicated that the gene expression of *A. niger* SICU-33 changed significantly with pH adjustment.

### 3.3 KEGG and GO enrichment analysis of DEGs

**Figure 4:**
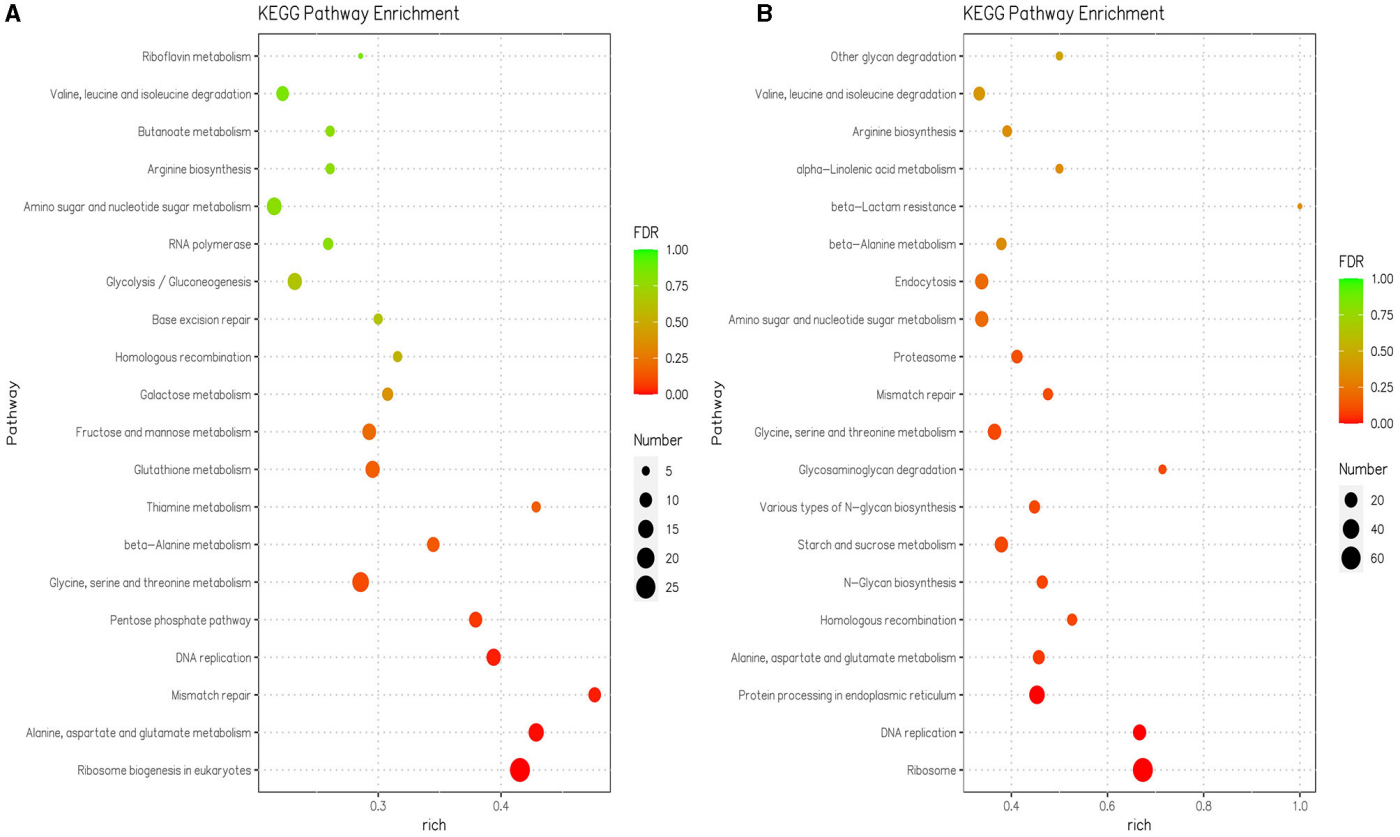
The 20 most significant KEGG pathways in the KEGG enrichment analysis were in pH 9 vs. pH 7 **(A)** and pH 5 vs. pH 7 **(B)** of *Aspergillus niger* SICU-33, respectively. Genes were considered differentially expressed when fold change (FC) ≥ 1.5 or ≤ 0.667 and *p* < 0.05.

In the KEGG enrichment analysis, the DEGs were assigned to 109 pathways at pH 5 compared with pH 7, and the DEGs were assigned to 105 pathways at pH 9 compared with pH 7 (Supplementary Table S2). KEGG pathways with significant enrichment (*p* < 0.05) of differentially expressed genes mainly include translation, amino acid metabolism, replication and repair, carbohydrate metabolism, amino acid metabolism, and metabolism of cofactors and vitamins in pH 9 compared with pH 7. The translation, replication and repair, folding, sorting and degradation, amino acid metabolism, glycan biosynthesis and metabolism, carbohydrate metabolism, transport and catabolism, and metabolism of other amino acids were enriched in pH 5 compared with pH 7 (Supplementary Table S2). The ribosome biogenesis in the eukaryotes pathway (ang03008) was the highest enriched at pH 9 and contained 27 downregulated DEGs (Figure 4A, Supplementary Table S2), where the *scaffold1.g1067* gene encodes an XRN1 protein with a domain similar to the 5′-3′ exoribonuclease and belongs to the exodeoxyribonucleases producing 5′-phosphomonoesters family; the *scaffold4.g566* encodes the RRM_SF protein and belongs to the RNA recognition motif (RRM) superfamily, the *scaffold6.g396* encodes the RIO1_euk protein, which belongs to the catalytic domain of the atypical protein serine kinase. There were 13 downregulated DEGs related to the biosynthesis and metabolism of glycan in pH 5 compared with pH 7, and these genes code for enzymes involved in carbohydrate metabolism, i.e., GH 47 (EC 3.2.1.24), GH 63N (EC 3.2.1.106), and ribophorin_I(EC:2.4.1.119). The pathways associated with genetic information processing included the ribosome (ang03010), which contained 66 upregulated DEGs in pH 5 compared with pH 7 (Figure 4B, Supplementary Table S2). Among them, *scaffold1.g1336* (CAK45721.1), *scaffold1.g820* (CAK37009.1), *scaffold1.g333* (CAK43220.1), s*caffold8.g739* (CAL00567.1), *scaffold2.g1279* (XP_020061647.1), and *scaffold4.g1193* (CAK38984.1) encodes different types of ribosomal protein, which plays an important role in the cellular ribosome and participates in protein synthesis.

**Figure 5:**
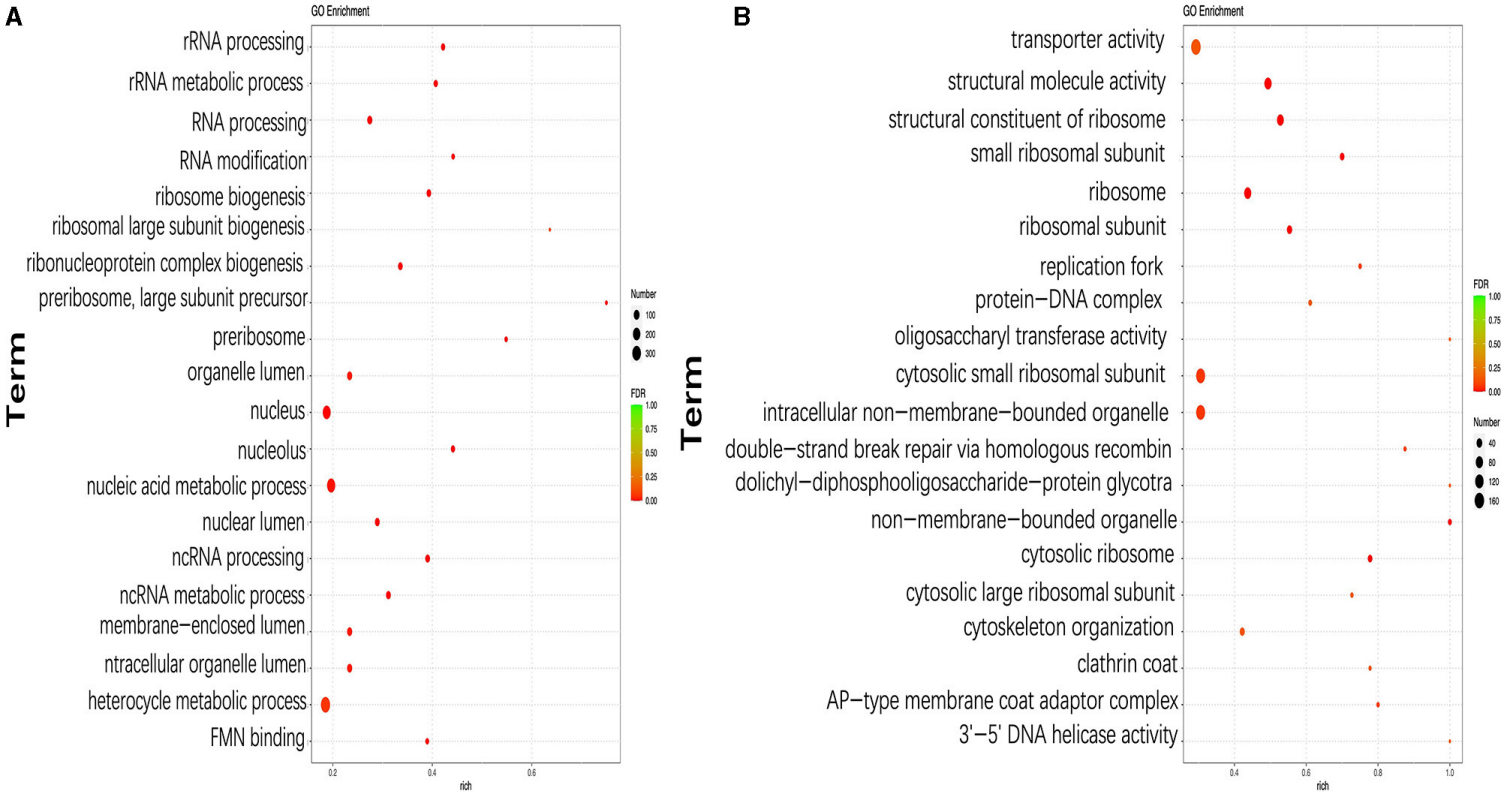
Top 20 most significantly enriched GO terms based on GO annotation of differentially expressed genes (DEGs) in pH 9 vs. pH 7 **(A)**, pH 5 vs. pH 7 **(B)** of *Aspergillus niger* SICU-33. Genes were considered differentially expressed when fold change (FC) ≥ 1.5 or ≤ 0.667 and *p* < 0.05.

The functions of DEGs were further explored using GO annotation analysis, and the results showed that the top three GO terms with the highest number of DEGs were the heterocycle metabolic process (GO:0046483; 115 upregulated and 234 downregulated), nucleus (GO:0090304; 69 upregulated and 192 downregulated), and nucleic acid metabolic process (GO:0005634; 61 upregulated and 175 downregulated) in pH 9 vs. pH7 (Figure 5A, Supplementary Table S3). In addition, DEGs in the following three GO terms of ribosomal large subunit biogenesis, preribosome, and preribosome, large subunit precursor were only downregulated, including six identical genes (*scaffold1.g583, scaffold2.g729, scaffold4.g751, scaffold5.g785, scaffold6.g295*, and *scaffold6.g908*) that appear in all above three GO terms (Supplementary Table S3). Moreover, we found that these six genes were involved in ribosome biosynthesis, and they encoded related proteins or provided precursor substances such as erb1 protein (A2QPZ4.1), brx1protein (GAQ33823.1), and MAK16 (CAL00962.1), etc. In pH 5 vs. pH 7, GO terms such as ribosome (GO:0005840), cytosolic ribosome (GO:0005840), cytosolic small ribosomal subunit (GO:0022627), small ribosomal subunit (GO:0015935), and ribosomal subunit (GO:0044391), including 158 DEGs were upregulated, indicating that ribosome metabolism of *Aspergillus niger* was more active at pH 5 (Figure 5B, Supplementary Table S3). Twelve genes appear together in all five of the above GO terms, and these genes also encode different types of ribosomal proteins similar to the KEGG results, i.e., 40S ribosomal protein S24 (CAK40980.1), 60S ribosomal protein L8 (CAK45721.1), and 40S ribosomal protein S7 (CAL00638.1).

### 3.4 qPCR validation of DEGs

The expression levels of 19 DEGs were randomly validated for real-time qPCR analysis. Among them, 17 genes passed the differential abundance criteria in both RNAseq and qPCR analysis (*p* < 0.05, Table 3), and the melting curve of the qPCR experiment is shown in Supplementary Figure S1: in pH 5 vs. pH 7, two out of seven genes assigned to the GO term are integral components of ribosome metabolism, three out of seven genes assigned to the GO term are related to carbohydrate metabolic processes, and three genes assigned to the GO term double-strand break repair via homologous recombination; In pH 9 vs. pH 7, five out of 10 genes assigned to the GO terms are related to nucleic acid metabolic process, and the remaining four genes are assigned to the GO terms with carbohydrate metabolic process. Specifically, the qPCR results revealed three genes, including *Scaffod1.g1067* (XRN1), *Scaffod4.g566* (RRM), and *Scaffod6.g396* (RIO1) were significantly downregulated at pH 9 relative to pH 7 (Supplementary Figure S2), which was consistent with the transcriptomic data.

**Table 3:** Validation by qRT-PCR of a total of 19 differentially expressed genes (DEGs) identified by transcriptome analysis of *Aspergillus niger* under group pH 5 vs. pH 7 and pH 9 vs. pH 7.

**Group**	**GO term**	**Gene ID**	**Gene name**	**RNA seq**		**qPCR**	
				**log**_2_ **(FC)**	***p*** **-value**	**log**_2_ **(FC)**	***p*** **-value**
pH 5 vs. pH 7	Structural constituent of ribosome	*Scaffold8.g739*	*An17g02240*	1.53	0.00^*^	1.68	0.04
	Ribosome	*Scaffold2.g820*	*An01g04740*	1.91	0.00^*^	1.87	0.03
	Carbohydrate metabolic process	*Scaffold6.g1141*	*ostA*	−1.55	0.00^*^	−1.59	0.01
		***Scaffold1.g1425***	***An06g01100***	**−1.41**	**0.00** ^*^	**−2.79**	**0.11**
		*Scaffold7.g486*	*An09g05880*	−1.44	0.00^*^	−1.94	0.02
		*Scaffold8.g662*	*An16g08570*	−2.41	0.00^*^	−2.03	0.01
	Double-strand break repair via homologous recombination	*Scaffold7.g389*	*An09g04640*	−1.59	0.00^*^	−1.42	0.03
		*Scaffold2.g970*	*An01g02720*	−1.45	0.00^*^	−1.51	0.01
		*Scaffold5.g1219*	*An15g07150*	−1.09	0.00^*^	−1.22	0.01
pH 9 vs. pH 7	Nucleic acid metabolic process	*Scaffold7.g1014*	*An14g05390*	−1.11	0.00^*^	−1.18	0.03
	Nucleic acid binding	*Scaffold3.g363*	*ANOM_001814*	−1.28	0.00^*^	−0.96	0.02
	ncRNA processing	*Scaffold1.g1067*	*An08g06790*	−1.09	0.00^*^	−1.27	0.01
	Nucleoside phosphate binding	*Scaffold4.g566*	*An04g00570*	−1.30	0.00^*^	−1.15	0.03
	Glucose metabolic process	*Scaffold4.g953*	*An04g05300*	1.81	0.00^*^	1.64	0.03
	Ribonucleotide binding	*Scaffold6.g396*	*An02g04830*	−1.45	0.00^*^	−1.83	0.02
	Carbohydrate metabolic process	***Scaffold6.g249***	***An02g02930***	**1.88**	**0.00** ^*^	**2.92**	**0.14**
		*Scaffold9.g46*	*ANOM_009576*	1.36	0.00^*^	1.73	0.02
	Polysaccharide catabolic process	*Scaffold4.g1067*	*aglU*	1.16	0.00^*^	1.44	0.01
	Ribose phosphate metabolic process	*Scaffold6.g1128*	*hxk*	1.77	0.00^*^	1.59	0.01

Two genes not verified by RT-qPCR are highlighted in bold. ^*^denote statistically significant differences (*P* < 0.05).

### 3.5 DEMs description of *Aspergillus niger*

**Figure 6:**
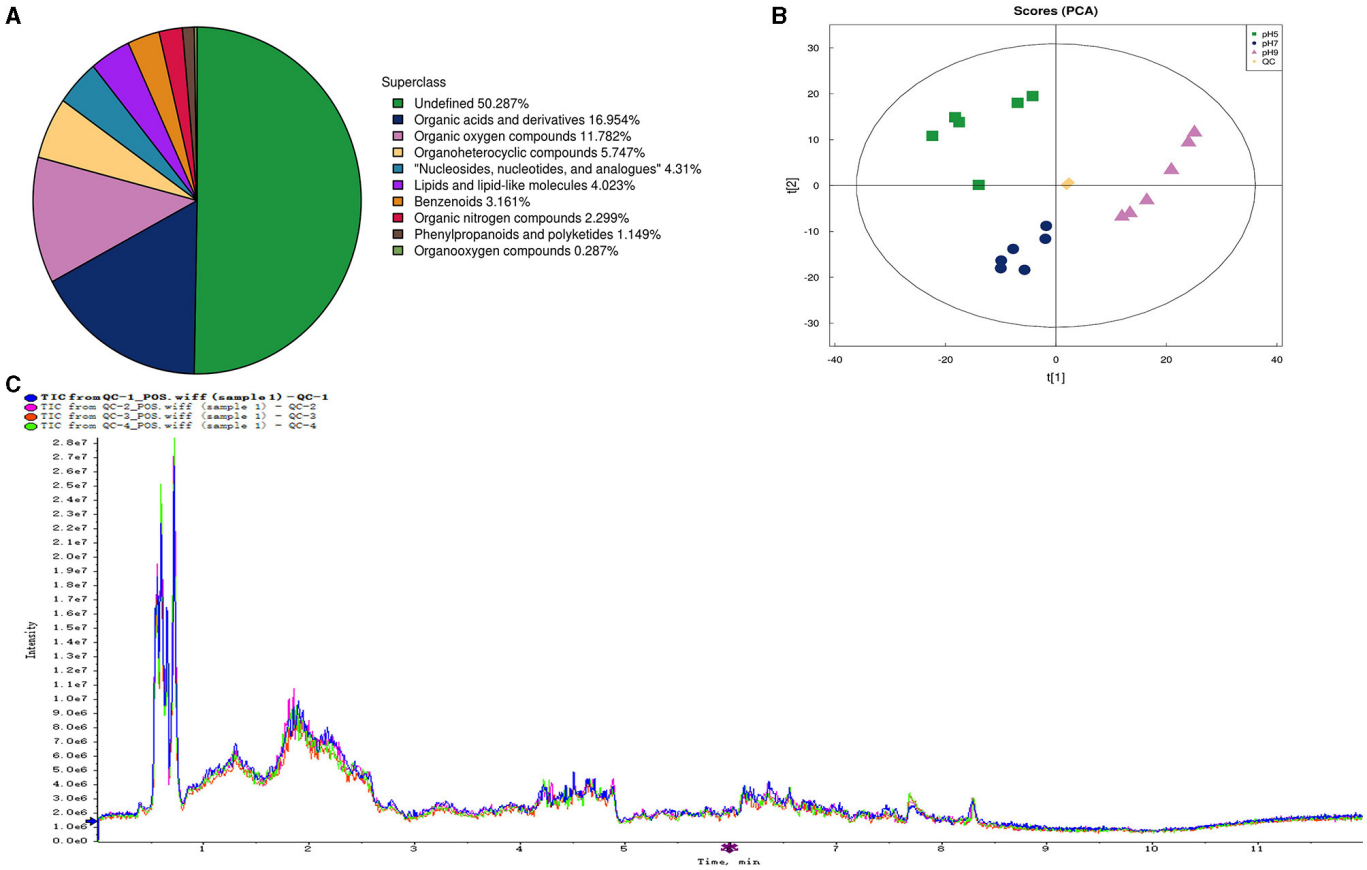
The number and proportion of metabolites identified in each chemical classification **(A)**. Principal component analysis (PCA) of the proteome samples of *Aspergillus niger* in positive ion patterns under different pH **(B)**. QC, quality control. Total Ion Chromatogram (TIC) of the proteome samples of *Aspergillus niger* in positive ion patterns under different pH **(C)**.

**Figure 7:**
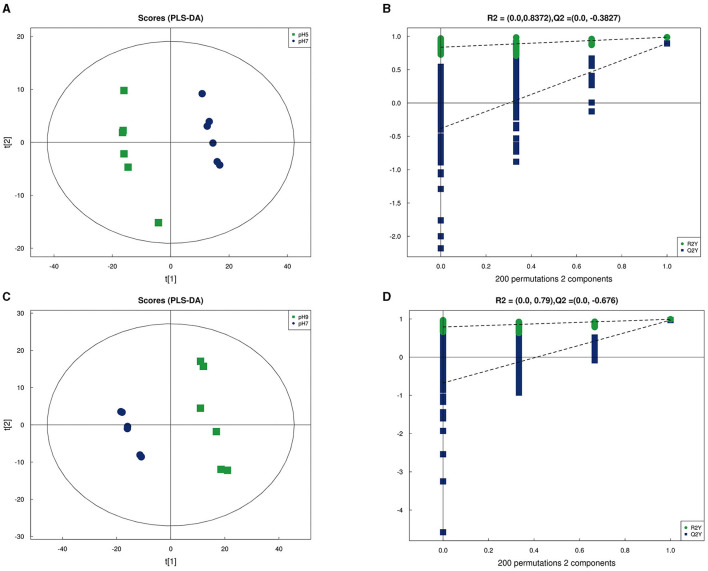
Partial Least Squares Discrimination Analysis (PLS-DA) in positive ion mode of *Aspergillus niger in* pH 5 vs. pH 7 **(A)** and pH 9 vs. pH 7 **(C)**; PLS-DA permutation test in pH 5 vs. pH 7 **(B)** and pH 9 vs. pH 7 **(D)**.

In our research, 348 metabolites were identified after the combination of positive and negative ion modes, and the proportion of various metabolites is shown in Figure 6A and Table 4, among which 236 metabolites were identified under the positive ion mode. In particular, the metabolites related to organic acids and derivatives accounted for the highest proportion of 16.954% (Figure 6A). PCA analysis (Figure 6B) and total ion chromatogram (TIC, Figure 6C) in positive ion patterns were performed to analyze the mass spectrometry data of the treatment group. The results showed that the response intensity and retention time of each chromatographic peak overlapped, and the six biological repeat data points in the treatment group could be collected together. The data points of the samples of the three varieties of the treatment groups could be clearly distinguished in space, indicating that the metabolites of each group of samples were different in terms of species, concentration, and quantity. In addition, the interpretation of the PLS-DA model was good; the samples were relatively dispersed among the groups, and the aggregation was obvious within the groups (Figures 7A, C). The permutation test results showed that *R*^2^ and *Q*^2^ of the random model gradually decreased with the gradual reduction of replacement retention, indicating that the model was reasonable and effective (Figures 7B, D).

**Table 4:** The number of metabolites identified in each chemical classification.

**Superclass**	**No. of identified metabolites**
Organic acids and derivatives	59
Organic oxygen compounds	41
Organoheterocyclic compounds	20
Nucleosides, nucleotides, and analogs	15
Lipids and lipid-like molecules	14
Benzenoids	11
Organic nitrogen compounds	8
Phenylpropanoids and polyketides	4
Organooxygen compounds	1
Undefined	175

**Figure 8:**
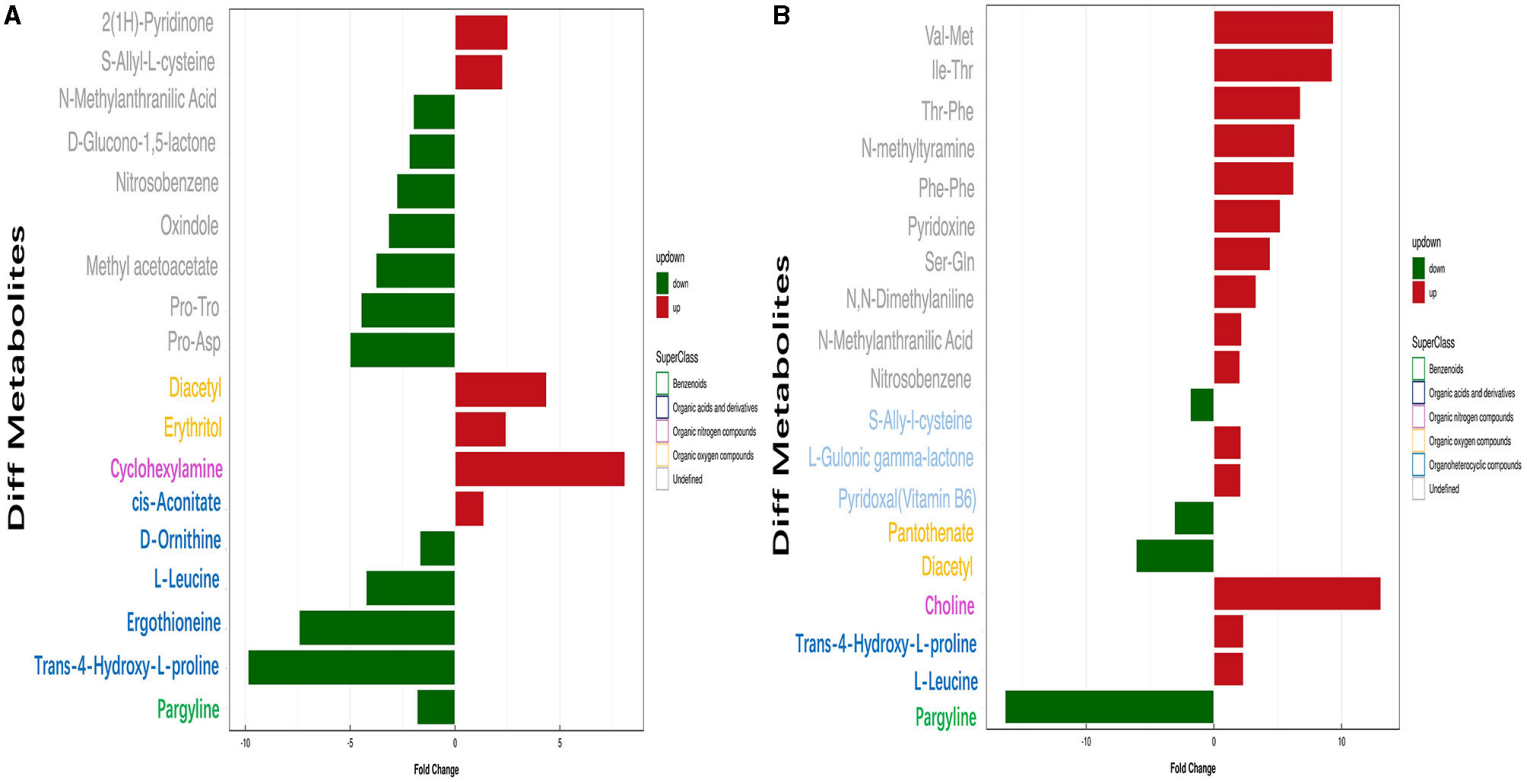
Analysis of significant differential metabolite expression in positive ion mode of *Aspergillus niger* SICU-33. **(A)** pH 5 vs. pH 7, **(B)** pH 9 vs. pH 7.

Conditions for screening differentially expressed metabolites (DEMs): (1) VIP ≥ 1; (2) *p*-value < 0.05. In two groups (pH 5 vs. pH 7 and pH 9 vs. pH 7), respectively, 19 and 20 significant DEMS were detected; most DEMs were uniquely associated with a specific pH treatment (Supplementary Table S4). Among the metabolites annotated based on chemical classification, four substances, including pargyline, trans-4-hydroxy-L-proline, ergothioneine, l-leucine, and d-ornithine, were downregulated at pH 5. Moreover, cyclohexylamine, diacetyl, and erythritol metabolites were upregulated at this pH value (Figure 8A). In pH 9 vs. pH 7, choline, trans-4-hydroxy-l-proline, and l-leucine were upregulated, but diacetyl was the opposite (Figure 8B). Interestingly, the change of pargyline at pH 9 is similar to pH 5, showing different degrees of downregulation.

**Figure 9:**
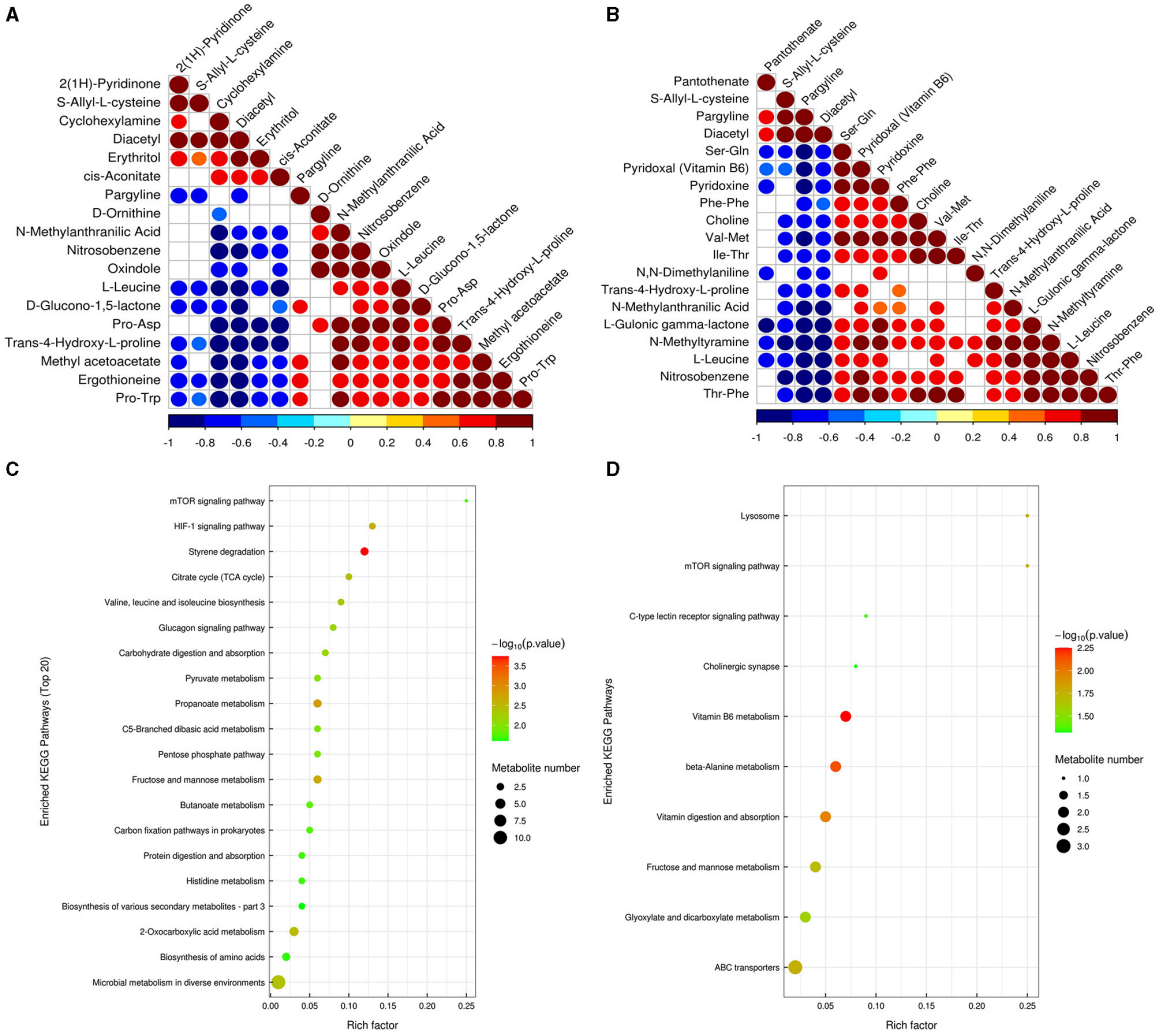
Correlation analysis of significant difference metabolites of *Aspergillus niger* SICU-33 in pH 5 vs. pH 7 **(A)** and pH 9 vs. pH 7 **(B)**; The significantly enriched KEGG pathways based on differentially expressed metabolites in pH 9 vs. pH7 and pH 5 vs. pH 7 of *Aspergillus niger* SICU-33.

Correlation analysis can help to measure the metabolic proximities of significantly different metabolites, which is conducive to further understanding the mutual regulatory relationship between metabolites in the process of biological state change. The results showed that there was a significant negative correlation between pargyline and diacetyl metabolites based on the metabolites annotated by the detection, indicating that there may be a synthetic transformation relationship between the above two substances at pH 5 (Figure 9A). In pH 9 vs. pH 7, choline and trans-4-hydroxy-l-proline were significantly negatively correlated with diacetyl and pargyline, indicating that the above four metabolites may synthesize and transform each other at pH 9 (Figure 9B). The enrichment analysis of the KEGG pathways showed that most of the DEMs were mainly enriched in carbohydrate metabolism; amino acid metabolism, biosynthesis of other secondary metabolites in pH 5 vs. pH 7, fructose and mannose metabolism (ko00051), and propanoate metabolism (ko00640) were the most significant carbohydrate metabolism pathways (Figure 9C, Supplementary Table S5). Specifically, DL-lactate was detected in two metabolic pathways associated with carbohydrates, and both were upregulated in pH 5 vs. pH 7 (Supplementary Table S6). Moreover, vitamin B6 metabolism (ko00750), beta-alanine metabolism (ko00410), and vitamin digestion and absorption (ko04977) were the top three significant metabolic pathways in pH 9 vs. pH 7 (Figure 9D, Supplementary Table S5). We also found that pyridoxal (vitamin B6) was upregulated in the corresponding metabolic process in pH 9 vs. pH 7 (Supplementary Table S6).

## 4 Discussion

### 4.1 Effects of pH change on physiological characteristics of *Aspergillus niger* SICU-33

Converting various organic and inorganic raw materials into valuable metabolites is a viable process in fungal systems, which offers great potential for the development of biotechnology production. For instance, filamentous fungi play an important role in the biological manufacture of primary and secondary metabolites (Meyer et al., 2020; Vorapreeda et al., 2021). During the process, microorganisms optimize growth conditions for their cellular functions. Metabolic disorders, and even cell death, can be caused by changes in the external environment, such as pH (Beales, 2004; Guan and Liu, 2020). More systematic studies on *A. niger* are needed to better understand the effects of pH on mycelial growth, biological metabolism, and polysaccharide metabolism. In the present study, the effect of different ambient pH on *A. niger* growth and biological synthesis process (*in vitro*) were studied. Our results show that, at pH 5 and pH 9, *A. niger* spore germination and mycelium biomass and growth were significantly inhibited, whereas the mycelium polysaccharide content reached the highest at pH 7. It has been reported that environmental pH can affect spore germination, transcriptional expression, and metabolism-related pathways of fungi pathogens (Li et al., 2010; Walaszczyk et al., 2018; Pang et al., 2020; Jimdjio et al., 2021). Relevant studies also showed that environmental burden had significant effects on the biochemical, macroscopic, and microscopic morphological characteristics of the strains (Šimonovičová et al., 2013). Furthermore, *A. niger* mycelium biomass and growth increased significantly at pH 7; the reason may be that strong acid or alkali conditions destroy the chromosome and protein, DNA structure, thus inhibiting the growth and metabolism of *A.niger* (Jimdjio et al., 2021). Some studies have shown that patulin was more stable at a pH range of 2.5 to 5.5 than at a more basic pH (Tannous et al., 2016). In our study, the content of polysaccharides in *A. niger* mycelium was correlated with pH. Similarly, Calvo et al. (2002) speculated that the production of aflatoxin and sterigmatocystin may be affected by the pH of the culture medium.

### 4.2 Ribosome biogenesis responds to pH changes of *Aspergillus niger* SICU-33

Previous studies have shown that pH value, as one of the important environmental parameters, has a crucial impact on the growth and metabolism of microorganisms (Li et al., 2010). Meanwhile, a few studies have also pointed out that some microorganisms can respond to the pH value of the growth environment and adjust the expression of genes in a certain pH range (Peñalva and Arst, 2002). For example, PacC was found to encode a transcription factor in *Aspergillus nidulans*, which showed activation under alkaline conditions and inhibition under acidic conditions, and related proteins were also confirmed to be associated with pH signal transduction and gene expression (Peñalva et al., 2008). In our study, KEGG analysis of the transcriptome showed that ribosome biogenesis in *A. niger* SICU-33 was more active at pH 9, which involved many biochemical reactions such as translation, protein modification, and carbohydrate metabolism. In particular, we found that the downregulated genes in this condition encode the XRN1 protein, RRM (RNA recognition motif) protein, and RIO1_euk protein, which is involved in translation while transmitting genetic information. Studies have shown that RRM was a very rich domain in eukaryotes and was present in proteins involved in RNA processing, export, and RNA stability; it is a key regulator of related metabolic processes in response to abiotic stress conditions in studies of plants (Ambrosone et al., 2012; Muthusamy et al., 2021).

Similarly, the zinc finger domain protein PacC in *Aspergillus nidulans* mediates the ambient pH regulatory pathway (Picazo et al., 2020); another RIO1 protein in our results is essential for eukaryotic survival and is required for 18S rRNA processing, normal cell cycle progression, and chromosome maintenance (LaRonde-LeBlanc and Wlodawer, 2005). In addition, Iacovella et al. (2018) found in their studies of *Saccharomyces cerevisiae* that the RIO1 protein directly regulates important signaling networks at the protein, chromatin, and RNA levels through a series of regulatory factors. Therefore, we speculate that RIO1 and RRM proteins in ribosome biogenesis may have a similar role in *Aspergillus niger*, responding to changes in external pH to maintain their normal metabolic processes, and alkaline conditions cause DEGs for both two proteins to be downregulated. However, ribosome biogenesis is still active, but genes encoding ribosome-related proteins are upregulated at pH 5, which seems to differ from the conclusion of alkaline conditions. The reason for this situation may be that this part of the protein has dual functions, which are acid-expressed and alkaline-expressed genes (Espeso and Arst, 2000). Acid-expressed genes are inhibited under alkaline conditions. Still, they positively regulate related genes under acidic conditions and acid and alkaline phosphatase in organisms illustrate this opposite effect (Caddick et al., 1986).

### 4.3 Characteristics of DEMs of *Aspergillus niger* SICU-33

Various studies have examined the metabolic network underlying aspergillus growth and metabolism using metabolomics (Xu et al., 2018; Li et al., 2019). In our study, several metabolites were found in different pH control groups. We noted that the pargyline was significantly downregulated at both acidic and alkaline conditions, while cyclohexylamine and choline were significantly upregulated at pH 5 and pH 9, respectively. It has been reported that the pargyline belongs to an inhibitor specific to flavin-containing amine oxidases in the study of filamentous fungi; the substance inhibits the activity of flavin-containing amine oxidases (EC 1.4.3.4.) (Frébort et al., 1997), which have been reported to be involved in oxidative metabolic processes in *Aspergillus niger* (Frederick et al., 2014). Therefore, under acidic and alkaline conditions, the downregulation of pargyline can lead to changes in the activity of flavin-containing amine oxidases, further affecting the oxidative metabolism of strain SICU-33.

Moreover, choline is negatively correlated with pargyline in alkaline conditions, indicating that there may be a relationship between the synthesis and transformation of these two substances in the metabolic process. Choline is an alkaloid that can be used as an enzyme inhibitor to combine with oxidase in cells, similar to pargyline (Luhová et al., 1996). In addition, D and L amino acids as external nitrogen sources have been shown to promote hyphal growth similar to D-ornithine and L-leucine (Mardon et al., 1969; Hara and Kino, 2020). Still, in our study, the expression of these two amino acids was downregulated by pH, indicating that the amino acid metabolism of *A.niger* may be inhibited under acidic or alkaline culture conditions. To sum up, pH affects the dissociation state of some nutrients in the medium, the physiological function of the cell membrane, and the activities of various enzymes in the cell, and then affects the growth of microorganisms and the synthesis of products (Guan and Liu, 2020).

### 4.4 Characteristics of carbohydrate metabolism with pH

Carbohydrate metabolism is an important process of microbial growth and development, and the accumulation of microbial nutrients can be improved by studying this process (Zhang et al., 2020). We found that the genes encoding GH 47, GH 63N, and ribophorin_I, which are related to the biosynthesis and metabolism of glycan, were downregulated under acidic conditions. These results suggest that acidic conditions may inhibit the expression of these genes and affect the normal carbohydrate metabolism of *Aspergillus niger*. In particular, ribophorin_I is an essential subunit of oligosaccharyltransferase (OST), which is also known as dolichyl-diphosphooligosaccharide-protein glycosyltransferase (EC: 2.4.1.119), catalyzing the transfer of oligosaccharides into the lumen of the endoplasmic reticulum (Pathak et al., 1995). In our analysis of DEMs related to carbohydrates, we found that DL-lactate was upregulated in acidic conditions, primarily in the fructose and mannose metabolism and propanoate metabolism metabolic pathways. Some studies have shown that lactic acid can inhibit the growth and metabolism of microorganisms due to its uncoupling and intracellular anion accumulation or chelation (Yáñez et al., 2008). Similarly, the accumulation of DL-lactate metabolites in our study may inhibit the growth of *Aspergillus niger*. In summary, ribophorin_I enzymes and DL-lactate may be important substances in response to pH changes during carbohydrate metabolism of *A. niger* SICU-33.

## 5 Conclusion

We used transcriptomic and metabolic methods to study *A.niger* cultured under different pH conditions (pH 5, pH 7, pH 9), focusing on ribosomal biogenesis and carbohydrate metabolism. The results showed that the biomass, growth rate, and polysaccharide content of *A.niger* in pH 7 were higher than those in acidic and alkaline medium. Transcriptome analysis showed that out of the 1,765 annotated genes, 450 and out of 1,516 annotated genes, 714 were differentially expressed in pH 9 vs. pH 7 and pH 5 vs. pH 9, respectively; DEGs related to ribosome biogenesis were the most abundant. The downregulated expression of genes encoding XRN1, RRM, and RIO1 affected protein translation, modification, and carbohydrate metabolism in fungi. The dynamic changes of pargyline and choline were in response to the oxidative metabolism of *A.niger*. The ribophorin_I enzymes and DL-lactate may be important substances related to pH changes during carbohydrate metabolism of *A.niger* SICU-33. In all, acidic and alkaline conditions can affect the growth and polysaccharide metabolism of *A.niger*, indicating that in the actual production and application of *A.niger*, in addition to the influence of environmental factors, we should also consider the change of related enzymes, the accumulation of enzyme inhibitors or increase the research on stress-tolerance related genes. As far as we know, this study revealed the molecular variation of *A.niger* at different pH through joint analysis of transcriptome and metabolome, providing a basic molecular mechanism for the identification of key genes of *A.niger* and further providing a strong experimental basis for the development and application of *A.niger* in agriculture.

## Data availability statement

The original contributions presented in the study are included in the article/Supplementary material, further inquiries can be directed to the corresponding author.

## Author contributions

RZ: Writing – review & editing, Writing – original draft, Validation, Formal analysis. YC: Writing – review & editing, Data curation, Formal analysis. WW: Writing – review & editing, Investigation, Formal analysis, Data curation. JC: Writing – review & editing, Resources, Investigation, Data curation. DL: Writing – review & editing, Supervision, Software, Formal analysis. LZ: Writing – review & editing, Methodology, Formal analysis, Data curation. QX: Writing – review & editing, Visualization, Software, Methodology, Formal analysis. KZ: Writing – review & editing, Software, Methodology, Formal analysis. MM: Writing – review & editing, Validation, Supervision, Formal analysis, Data curation. XY: Writing – review & editing, Formal analysis, Software, Methodology. QC: Writing – review & editing, Validation, Methodology, Conceptualization. PP: Formal analysis, Writing – review & editing, Software, Methodology. YG: Software, Resources, Project administration, Methodology, Funding acquisition, Conceptualization, Writing – review & editing, Writing – original draft.

## References

[B1] Abdel-Azeem A. M. Abdel-Azeem M. A. Abdul-Hadi S. Y. Darwish A. G. (2019). “*Aspergillus*: biodiversity, ecological significances, and industrial applications,” in Recent Advancement in White Biotechnology. Through Fungi. Fungal Biology, Vol. 1, eds. A. Yadav, S. Mishra, S. Singh, and A. Gupta (Cham: Springer), 121–179. 10.1007/978-3-030-10480-1_4

[B2] Altschul S. F. Madden T. L. Schäffer A. A. Zhang J. Zheng Zhang Z. Miller W.. (1997). Gapped BLAST and PSI-BLAST: a new generation of protein database search programs. Nucleic Acids Res. 25, 3389–3402. 10.1093/nar/25.17.3389 9254694 PMC146917

[B3] Ambrosone A. Costa A. Leone A. Grillo S. (2012). Beyond transcription: RNA-binding proteins as emerging regulators of plant response to environmental constraints. Plant Sci. 182, 12–18. 10.1016/j.plantsci.2011.02.004 22118611

[B4] Andersen M. R. Nielsen M. L. Nielsen J. (2008). Metabolic model integration of the bibliome, genome, metabolome and reactome of *Aspergillus niger*. Mol. Syst. Biol. 4:178. 10.1038/msb.2008.12 18364712 PMC2290933

[B5] Apweiler R. Bairoch A. Wu C. H. Barker W. C. Boeckmann B. Ferro S.. (2004). UniProt: the universal protein knowledgebase. Nucleic Acids Res. 32, D115–D119. 10.1093/nar/gkh131 14681372 PMC308865

[B6] Ashburner M. Ball C. A. Blake J. A. Botstein D. Butler H. Cherry J. M.. (2000). Gene ontology: tool for the unification of biology. The Gene Ontology Consortium. Nat. Genet. 25, 25–29. 10.1038/75556 10802651 PMC3037419

[B7] Basu S. Bose C. Ojha N. Das N. Das J. Pal M.. (2015). Evolution of bacterial and fungal growth media. Bioinformation 11, 182–184. 10.6026/97320630011182 26124557 PMC4479053

[B8] Beales N. (2004). Adaptation of microorganisms to cold temperatures, weak acid preservatives, low pH, and osmotic stress: a review. Compr. Rev. Food Sci. Food Saf. 3, 1–20. 10.1111/j.1541-4337.2004.tb00057.x 33430556

[B9] Boecker S. Grätz S. Kerwat D. Adam L. Schirmer D. Richter L.. (2018). *Aspergillus niger* is a superior expression host for the production of bioactive fungal cyclodepsipeptides. Fungal Biol Biotechnol. 5:4. 10.1186/s40694-018-0048-3 29507740 PMC5833056

[B10] Caddick M. X. Brownlee A. G. Arst H. N. (1986). Regulation of gene expression by pH of the growth medium in *Aspergillus nidulans*. Mol. Gen. Genet. 203, 346–353. 10.1007/BF00333978 3016485

[B11] Calvo A. M. Wilson R. A. Bok J. W. Keller N. P. (2002). Relationship between secondary metabolism and fungal development. Microbiol. Mol. Biol. Rev. 66, 447–459. 10.1128/MMBR.66.3.447-459.2002 12208999 PMC120793

[B12] Chen X. Mao X. Huang J. Ding Y. Wu J. Dong S.. (2011). KOBAS 2.0: a web server for annotation and identification of enriched pathways and diseases. Nucleic Acids Res. 39, W316–W322. 10.1093/nar/gkr483 21715386 PMC3125809

[B13] Chen Y. Li E. M. Xu L. Y. (2022b). Guide to metabolomics analysis: a bioinformatics workflow. Metabolites 12:357. 10.3390/metabo12040357 35448542 PMC9032224

[B14] Chen Y. Liang J. Zia A. Gao X. Wang Y. Zhang L.. (2022a). Culture dependent and independent characterization of endophytic bacteria in the seeds of highland barley. Front. Microbiol. 13:981158. 10.3389/fmicb.2022.981158 36246264 PMC9555213

[B15] Coleine C. Stajich J. Selbmann L. (2022). Fungi are key players in extreme ecosystems. Trends Ecol. Evol. 37, 517–528. 10.1016/j.tree.2022.02.002 35246323

[B16] Dawson I. K. Russell J. Powell W. Steffenson B. Thomas W. T. B. Waugh R.. (2015). Barley: a translational model for adaptation to climate change. New Phytol. 206, 913–931. 10.1111/nph.13266 25605349

[B17] Devi R. Kaur T. Kour D. Rana K. L. Yadav A. Yadav A. N.. (2020). Beneficial fungal communities from different habitats and their roles in plant growth promotion and soil health. Microb Biosyst. 5, 21–47. 10.21608/mb.2020.32802.1016

[B18] Dou X. Zhou Z. Zhao L. (2021). Identification and expression analysis of miRNAs in germination and seedling growth of Tibetan hulless barley. Genomics 113, 3735–3749. 10.1016/j.ygeno.2021.08.019 34517091

[B19] Espeso E. A. Arst Jr H. N. (2000). On the mechanism by which alkaline pH prevents expression of an acid-expressed gene. Mol. Cell. Biol. 20, 3355–3363. 10.1128/MCB.20.10.3355-3363.2000 10779325 PMC85628

[B20] Frébort I. Matsushita K. Adachi O. (1997). Involvement of multiple copper/topa quinone-containing and flavin-containing amine oxidases and NAD(P)+ aldehyde dehydrogenases in amine degradation by filamentous fungi. J. Ferment. Bioeng. 84, 200–212. 10.1016/S0922-338X(97)82055-7

[B21] Frederick R. E. Ojha S. Lamb A. DuBois J. L. (2014). How pH modulates the reactivity and selectivity of a siderophore-associated flavin monooxygenase. Biochem 53, 2007–2016. 10.1021/bi401256b 24490904 PMC3985866

[B22] Glickmann E. Dessaux Y. (1995). A critical examination of the specificity of the salkowski reagent for indolic compounds produced by phytopathogenic bacteria. Appl. Environ. Microbiol. 61, 793–796. 10.1128/aem.61.2.793-796.1995 16534942 PMC1388360

[B23] Guan N. Liu L. (2020). Microbial response to acid stress: mechanisms and applications. Appl. Microbiol. Biotechnol. 104, 51–65. 10.1007/s00253-019-10226-1 31773206 PMC6942593

[B24] Hadgu K. Kooistra L. Rossing W. van Bruggen A. (2009). Assessing the effect of *Faidherbia albida* based land use systems on barley yield at field and regional scale in the highlands of Tigray, Northern Ethiopia. Food Secur. 1, 337–350. 10.1007/s12571-009-0030-2

[B25] Hara R. Kino K. (2020). Enzymatic reactions and microorganisms producing the various isomers of hydroxyproline. Appl. Microbiol. Biotechnol. 104, 4771–4779. 10.1007/s00253-020-10603-1 32291491

[B26] Hawksworth D. (2001). The magnitude of fungal diversity: the 1.5 million species estimate revisited. Mycol. Res. 105, 1422–1432. 10.1017/S095375620100472528752818

[B27] Hossain A. Hendrikx A. Punt P. (2019). Identification of novel citramalate biosynthesis pathways in *Aspergillus niger*. Fungal Biol Biotechnol. 6:19. 10.1186/s40694-019-0084-7 31827810 PMC6862759

[B28] Huang X. (2020). R Zhang, Y Qiu, H Wu, Q Xian, X Yu, et al. RNA-seq profiling showed divergent carbohydrate-active enzymes (CAZymes) expression patterns in *Lentinula edodes* at brown film formation stage under blue light induction. Front. Microbiol. 11:1044. 10.3389/fmicb.2020.01044 32536907 PMC7267012

[B29] Huerta-Cepas J. Szklarczyk D. Forslund K. Cook H. Heller D. Walter M. C.. (2016). eggNOG 4.5: a hierarchical orthology framework with improved functional annotations for eukaryotic, prokaryotic and viral sequences. Nucleic Acids Res. 44, D286–D293. 10.1093/nar/gkv1248 26582926 PMC4702882

[B30] Iacovella M. G. Bremang M. Basha O. Giacò L. Carotenuto W. Golfieri C.. (2018). Integrating Rio1 activities discloses its nutrient-activated network in *Saccharomyces cerevisiae*. Nucleic Acids Res. 46, 7586–7611. 10.1093/nar/gky618 30011030 PMC6125641

[B31] Jain K. K. Kumar A. Shankar A. Pandey D. Chaudhary B. Sharma K. K.. (2020). De novo transcriptome assembly and protein profiling of copper-induced lignocellulolytic fungus *Ganoderma lucidum* MDU-7 reveals genes involved in lignocellulose degradation and terpenoid biosynthetic pathways. Genomics 112, 184–198. 10.1016/j.ygeno.2019.01.012 30695716

[B32] Jimdjio C. K. Xue H. Bi Y. Nan M. Li L. Zhang R.. (2021). Effect of ambient pH on growth, pathogenicity, and patulin production of *Penicillium expansum*. Toxins 13:550. 10.3390/toxins13080550 34437421 PMC8402621

[B33] Jin N. Liu S. Peng H. Huang W. Kong L. Peng D.. (2021). Effect of *Aspergillus niger* NBC001 on the soybean rhizosphere microbial community in a soybean cyst nematode-infested field. J Integr Agr. 20, 3230–3239. 10.1016/S2095-3119(20)63467-0

[B34] Kadri T. Rouissi T. Brar S. K. Cledon M. Sarma S. Verma M.. (2017). Biodegradation of polycyclic aromatic hydrocarbons (PAHs) by fungal enzymes: a review. J. Environ. Sci. 51, 52–74. 10.1016/j.jes.2016.08.023 28115152

[B35] Kanehisa M. Goto S. Kawashima S. Okuno Y. Hattori M. (2004). The KEGG resource for deciphering the genome. Nucleic Acids Res. 32, D277–D280. 10.1093/nar/gkh063 14681412 PMC308797

[B36] Koza N. A. Adedayo A. A. Babalola O. O. Kappo A. P. (2022). Microorganisms in plant growth and development: roles in abiotic stress tolerance and secondary metabolites secretion. Microorganisms 10:1528. 10.3390/microorganisms10081528 36013946 PMC9415082

[B37] Krijgsheld P. Bleichrodt P. Van Veluw G. J. Wang F. Müller W. H. Dijksterhuis J.. (2013). Development in *Aspergillus*. Stud. Mycol. 74, 1–29. 10.3114/sim0006 23450714 PMC3563288

[B38] Lammirato C. Miltner A. Kaestner M. (2011). Effects of wood char and activated carbon on the hydrolysis of cellobiose by β-glucosidase from *Aspergillus niger*. Soil Biol. Biochem. 43, 1936–1942. 10.1016/j.soilbio.2011.05.021

[B39] LaRonde-LeBlanc N. Wlodawer A. (2005). The RIO kinases: an atypical protein kinase family required for ribosome biogenesis and cell cycle progression. BBA Proteins Proteom. 1754, 14–24. 10.1016/j.bbapap.2005.07.037 16182620

[B40] Li A. van Luijk N. (2011). M ter Beek, M Caspers, P Punt, M van der Werf. A clone-based transcriptomics approach for the identification of genes relevant for itaconic acid production in *Aspergillus*. Fungal Genetics Biol. 48, 602–611. 10.1016/j.fgb.2011.01.013 21324422

[B41] Li B. Lai T. Qin G. Tian S. (2010). Ambient pH stress inhibits spore germination of *Penicillium expansum* by impairing protein synthesis and folding: a proteomic-based study. J. Proteome Res. 9, 298–307. 10.1021/pr900622j 19951004

[B42] Li X. Pan L. Wang B. Pan L. (2019). The histone deacetylases hosa and hdaa affect the phenotype and transcriptomic and metabolic profiles of *Aspergillus niger*. Toxins 11:520. 10.3390/toxins11090520 31500299 PMC6784283

[B43] Love M. I. Huber W. Anders S. (2014). Moderated estimation of fold change and dispersion for RNA-seq data with DESeq2. Genome Biol. 15, 1–21. 10.1186/s13059-014-0550-8 25516281 PMC4302049

[B44] Luhová L. Slavík L. E. Frébort I. Sebela M. Zajoncová L. Peč P.. (1996). Comparison of kinetic properties between plant and fungal amine oxidases. J. Enzym. Inhib. 10, 251–262. 10.3109/14756369609036532 8872745

[B45] Ma Y. Ling T. Su X. Jiang B. Nian B. Chen L.. (2021). Integrated proteomics and metabolomics analysis of tea leaves fermented by *Aspergillus niger, Aspergillus tamarii* and *Aspergillus fumigatus*. Food Chem. 334:127560. 10.1016/j.foodchem.2020.127560 32711271

[B46] Mahadevamurthy M. Channappa T. M. Sidappa M. Raghupathi M. S. Nagaraj A. K. (2016). Isolation of phosphate solubilizing fungi from rhizosphere soil and its effect on seed growth parameters of different crop plants. J Appl Biol Biotechnol. 4, 022–026. 10.7324/JABB.2016.4060438341878

[B47] Manteau S. Abouna S. Lambert B. Legendre L. (2003). Differential regulation by ambient pH of putative virulence factor secretion by the phytopathogenic fungus *Botrytis cinerea*. FEMS Microbiol. Ecol. 43, 359–366. 10.1111/j.1574-6941.2003.tb01076.x 19719667

[B48] Mardon D. Balish E. Phillips A. W. (1969). Control of dimorphism in a biochemical variant of *Candida albicans*. J. Bacteriol. 100, 701–707. 10.1128/jb.100.2.701-707.1969 5354942 PMC250147

[B49] Marzo C. Díaz A. Caro I. Blandino A. (2019). Valorization of agro-industrial wastes to produce hydrolytic enzymes by fungal solid-state fermentation. Waste Manage Res. 37, 149–156. 10.1177/0734242X18798699 30222065

[B50] Meyer V. Basenko E. Y. Benz J. P. Braus G. H. Caddick M. X. Csukai M.. (2020). Growing a circular economy with fungal biotechnology: a white paper. Fungal Biol Biotechnol. 7, 1–23. 10.1186/s40694-020-00095-z 32280481 PMC7140391

[B51] Mi H. Muruganujan A. Huang X. Ebert D. Mills C. Guo X.. (2019). Protocol update for large-scale genome and gene function analysis with the PANTHER classification system (v. 14.0). Nat Protoc. 14, 703–721. 10.1038/s41596-019-0128-8 30804569 PMC6519457

[B52] Muthusamy M. Kim J. H. Kim J. A. Lee S. I. (2021). Plant RNA binding proteins as critical modulators in drought, high salinity, heat, and cold stress responses: an updated overview. Int. J. Mol. Sci. 22:6731. 10.3390/ijms22136731 34201749 PMC8269355

[B53] Nie C. Li T. Fan M. Wang Y. Sun Y. He R.. (2022). Polyphenols in highland barley tea inhibit the production of advanced glycosylation end-products and alleviate the skeletal muscle damage. Mol. Nutr. Food Res. 66:2200225. 10.1002/mnfr.202200225 35894228

[B54] Obadi M. Yajing Q. Bin X. (2021). Highland barley starch (Qingke): structures, properties, modifications, and applications. Int. J. Biol. Macromol. 185, 725–738. 10.1016/j.ijbiomac.2021.06.204 34224757

[B55] Ozsolak F. Milos P. M. (2011). RNA sequencing: advances, challenges and opportunities. Nat. Rev. Genet. 12, 87–98. 10.1038/nrg2934 21191423 PMC3031867

[B56] Pang K. L. Chiang M. W. Guo S. Y. Shih C. Y. Dahms H. U. Hwang J. S.. (2020). Growth study under combined effects of temperature, pH and salinity and transcriptome analysis revealed adaptations of *Aspergillus terreus* NTOU4989 to the extreme conditions at Kueishan Island Hydrothermal Vent Field, Taiwan. PLoS ONE 15:e0233621. 10.1371/journal.pone.0233621 32453769 PMC7250430

[B57] Pathak R. Parker C. S. Imperiali B. (1995). The essential yeast NLT1 gene encodes the 64 kDa glycoprotein subunit of the oligosaccharyl transferase. FEBS Lett. 362, 229–234. 10.1016/0014-5793(95)00253-6 7720878

[B58] Pel H. J. Winde J. d. e. Archer D. Dyer D. Hofmann G. Schaap P.. (2007). Genome sequencing and analysis of the versatile cell factory *Aspergillus niger* CBS 513.88. Nat. Biotechnol. 25, 221–31. 10.1038/nbt1282 17259976

[B59] Peñalva M. A. Arst Jr H. N. (2002). Regulation of gene expression by ambient pH in filamentous fungi and yeasts. Microbiol. Mol. Biol. Rev. 66, 426–446. 10.1128/MMBR.66.3.426-446.2002 12208998 PMC120796

[B60] Peñalva M. A. Tilburn J. Bignell E. Arst Jr H. N. (2008). Ambient pH gene regulation in fungi: making connections. Trends Microbiol. 16, 291–300. 10.1016/j.tim.2008.03.006 18457952

[B61] Picazo I. Etxebeste O. Requena E. Garzia A. Espeso E. A. (2020). Defining the transcriptional responses of *Aspergillus nidulans* to cation/alkaline pH stress and the role of the transcription factor SltA. Microb Genom. 6:e000415. 10.1099/mgen.0.000415 32735212 PMC7641419

[B62] Prusky D. B. Sionov E. (2021). Special Issue “Interplay between fungal pathogens and harvested crops and fruits”. Microorganisms 9, 553. 10.3390/microorganisms9030553 33800331 PMC7998692

[B63] Qiu H. Ge T. Liu J. Chen X. Hu Y. Wu J.. (2018). Effects of biotic and abiotic factors on soil organic matter mineralization: experiments and structural modeling analysis. Euro. J. Soil Biol. 84, 27–34. 10.1016/j.ejsobi.2017.12.00336481133

[B64] Qu Q. Wang L. Li L. He Y. Yang M. Ding Z. (2015). Effect of the fungus, *Aspergillus niger*, on the corrosion behaviour of AZ31B magnesium alloy in artificial seawater. Corros. Sci. 98, 249–259. 10.1016/j.corsci.2015.05.038

[B65] Schuster E. Dunn-Coleman N. Frisvad J. van Dijck P. (2002). On the safety of *Aspergillus niger* – a review. Appl. Microbiol. Biotechnol. 59, 426–435. 10.1007/s00253-002-1032-6 12172605

[B66] Schwyn B. Neilands J. B. (1987). Universal chemical assay for the detection and determination of siderophores. Anal. Biochem.,160, 47–56. 10.1016/0003-2697(87)90612-9 2952030

[B67] Sharma K. K. (2016). Fungal genome sequencing: basic biology to biotechnology. Crit. Rev. Biotechnol. 36, 743–759. 10.3109/07388551.2015.1015959 25721271

[B68] Silveira V. D. C. Pinto M. M. Westphal F. S. (2019). Influence of environmental factors favorable to the development and proliferation of mold in residential buildings in tropical climates. Build. Environ. 166, 106421. 10.1016/j.buildenv.2019.106421

[B69] Šimonovičová A. Hlinková E. Chovanová K. Pangallo D. (2013). Influence of the environment on the morphological and biochemical characteristics of different *Aspergillus niger* wild type strains. Indian J. Microbiol. 53, 187–193. 10.1007/s12088-012-0317-4 24426107 PMC3626960

[B70] Smith C. A. Want E. J. (2006). GO'Maille, R Abagyan, G Siuzdak. XCMS: processing mass spectrometry data for metabolite profiling using nonlinear peak alignment, matching, and identification. Anal. Chem. 78, 779–787. 10.1021/ac051437y 16448051

[B71] Sun R. Yi Z. Fu Y. Liu H. (2022). Dynamic changes in rhizosphere fungi in different developmental stages of wheat in a confined and isolated environment. Appl. Microbiol. Biotechnol. 106, 441–453. 10.1007/s00253-021-11698-w 34870738

[B72] Tai B. Chang J. Liu Y. Xing F. (2020). Recent progress of the effect of environmental factors on *Aspergillus flavus* growth and aflatoxins production on foods. Food Qual Saf. 4, 21–28. 10.1093/fqsafe/fyz040

[B73] Takano T. Yamamoto N. Suzuki T. Dohra H. Choi J. H. Terashima Y.. (2019). Genome sequence analysis of the fairy ring-forming fungus *Lepista sordida* and gene candidates for interaction with plants. Sci. Rep. 9:5888. 10.1038/s41598-019-42231-9 30971747 PMC6458111

[B74] Tannous J. Atoui A. El Khoury A. Francis Z. Oswald I. P. Puel O.. (2016). A study on the physicochemical parameters for *Penicillium expansum* growth and patulin production: effect of temperature, pH, and water activity. Food Sci Nutr. 4, 611–622. 10.1002/fsn3.324 27386110 PMC4930504

[B75] Teather R. M. Wood P. J. (1982). Use of Congo red-polysaccharide interactions in enumeration and characterization of cellulolytic bacteria from the bovine rumen. Appl. Environ. Microbiol. 43, 777–780. 10.1128/aem.43.4.777-780.1982 7081984 PMC241917

[B76] Tong Z. Zheng X. Tong Y. Shi Y. Sun J. (2019). Systems metabolic engineering for citric acid production by *Aspergillus niger* in the post-genomic era. Microb. Cell Fact. 18, 1–15. 10.1186/s12934-019-1064-6 30717739 PMC6362574

[B77] Vorapreeda T. Khongto B. Thammarongtham C. Srisuk T. Laoteng K. (2021). Metabolic regulation of sugar assimilation for lipid production in *Aspergillus oryzae* BCC7051 through comparative transcriptome perspective. Biology 10:885. 10.3390/biology10090885 34571762 PMC8467706

[B78] Walaszczyk E. Podgorski W. Janczar-Smuga M. Dymarska E. (2018). Effect of medium pH on chemical selectivity of oxalic acid biosynthesis by *Aspergillus niger* W78C in submerged batch cultures with sucrose as a carbon source. Chem. Zvesti 72, 1089–1093. 10.1007/s11696-017-0354-x 29681682 PMC5908826

[B79] Wang L. Feng Z. Wang X. Wang X. Zhang X. (2010). DEGseq: an R package for identifying differentially expressed genes from RNA-seq data. Bioinformatics 26, 136–138. 10.1093/bioinformatics/btp612 19855105

[B80] Wang Z. Gerstein M. Snyder M. (2009). RNA-Seq: a revolutionary tool for transcriptomics. Nat. Rev. Genet. 10, 57–63. 10.1038/nrg2484 19015660 PMC2949280

[B81] Xie H. Ma Q. Wei D. Wang F. (2018). Transcriptomic analysis of *Aspergillus niger* strains reveals the mechanism underlying high citric acid productivity. Bioresour. Bioprocess. 5:21. 10.1186/s40643-018-0208-6

[B82] Xu Q. Fu Y. Li S. Jiang L. Guan R. Huang H.. (2018). Integrated transcriptomic and metabolomic analysis of *Rhizopus oryzae* with different morphologies. Process Biochem. 64, 74–82. 10.1016/j.procbio.2017.10.001

[B83] Xue Z. Wang B. Qu C. Tao M. Wang Z. Zhang G.. (2023). Response of salt stress resistance in highland barley (*Hordeum vulgare* L. var. nudum) through phenylpropane metabolic pathway. PLoS ONE 18:e0286957. 10.1371/journal.pone.0286957 37788272 PMC10547159

[B84] Yáñez R. Marques S. Gírio F. M. Roseiro J. C. (2008). The effect of acid stress on lactate production and growth kinetics in *Lactobacillus rhamnosus* cultures. Process Biochem. 43, 356–361. 10.1016/j.procbio.2007.12.014

[B85] Zeng X. Guo Y. Xu Q. Mascher M. Guo G. Li S.. (2018). Origin and evolution of qingke barley in Tibet. Nat. Commun. 9:5433. 10.1038/s41467-018-07920-5 30575759 PMC6303313

[B86] Zhang C. Gao Z. Shi W. Li L. Tian R. Huang J.. (2020). Material conversion, microbial community composition and metabolic functional succession during green soybean hull composting. Bioresou r.Technol. 316:123823. 10.1016/j.biortech.2020.123823 32795866

