# Supplementary material for: Combined transcriptomic and metabolomic analysis revealed that pH changes affected the expression of carbohydrate and ribosome biogenesis-related genes in Aspergillus niger SICU-33
Source: Front Microbiol. 2024 Jun 19;15:1389268. doi: 10.3389/fmicb.2024.1389268 (PMC11220263; doi:10.3389/fmicb.2024.1389268)
Supplement: Supplementary file 2 [file Data_Sheet_1.docx]

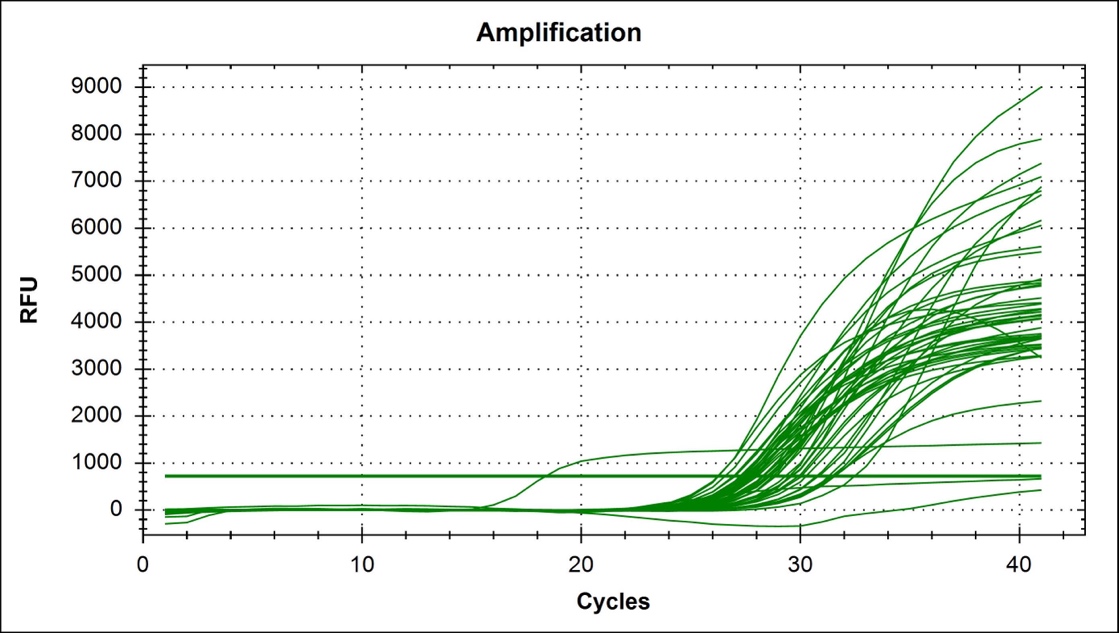


**Figure S1.** The melting curves of differentially expressed genes were verified by RT-qPCR.


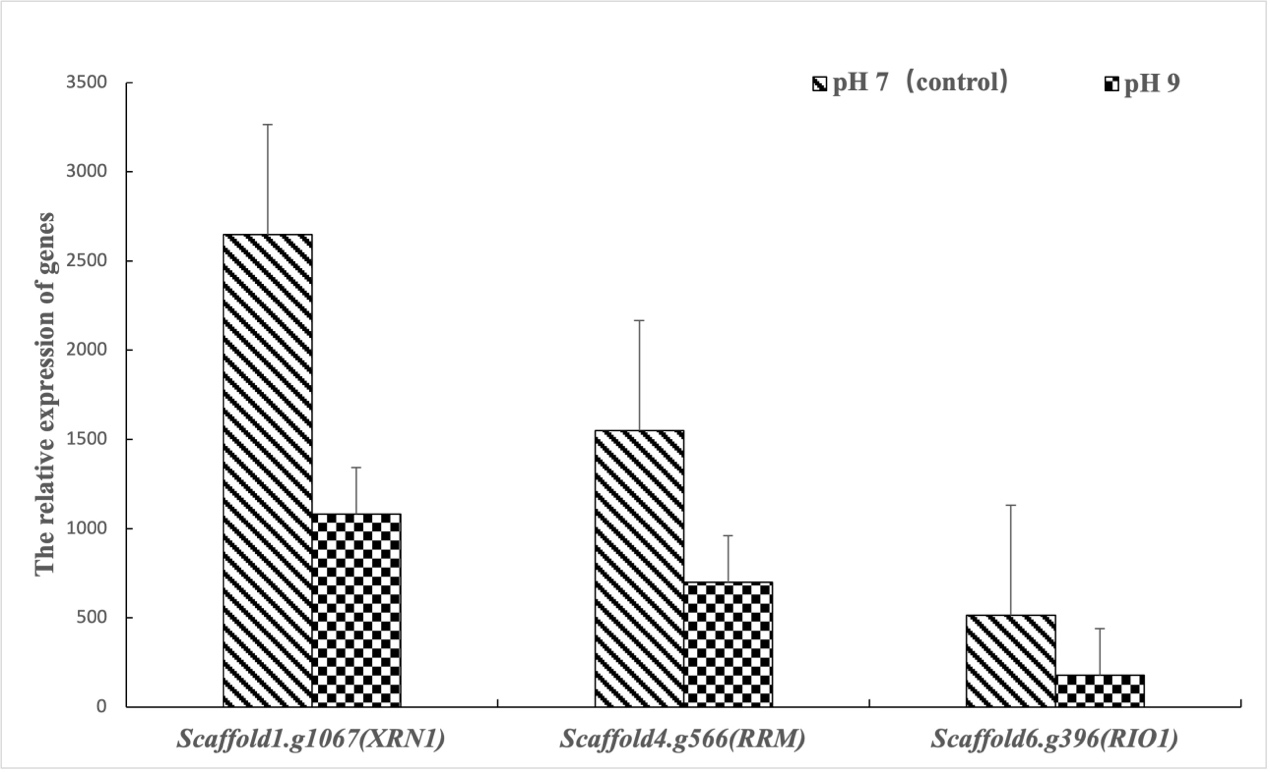


**Figure S2.** The relative expression of three DEGs including *Scaffod1.g1067* (XRN1), *Scaffod4.g566* (RRM), *Scaffod6.g396* (RIO1) verified by RT-qPCR of *A. niger* under group pH 9.0 vs. pH 7.0.
